# *Salmonella* Effector SteE Converts the Mammalian Serine/Threonine Kinase GSK3 into a Tyrosine Kinase to Direct Macrophage Polarization

**DOI:** 10.1016/j.chom.2019.11.002

**Published:** 2020-01-08

**Authors:** Ioanna Panagi, Elliott Jennings, Jingkun Zeng, Regina A. Günster, Cullum D. Stones, Hazel Mak, Enkai Jin, Daphne A.C. Stapels, Nur.Z. Subari, Trung H.M. Pham, Susan M. Brewer, Samantha Y.Q. Ong, Denise M. Monack, Sophie Helaine, Teresa L.M. Thurston

**Affiliations:** 1 MRC Centre for Molecular Bacteriology and Infection, Imperial College London, London, UK; 2 Departments of Microbiology and Immunology, Stanford University, Stanford, CA, USA

**Keywords:** macrophage polarization, M2, *Salmonella*, SteE, STAT3, SPI2, GSK3, dual-specificity kinases, host-pathogen interactions

## Abstract

Many Gram-negative bacterial pathogens antagonize anti-bacterial immunity through translocated effector proteins that inhibit pro-inflammatory signaling. In addition, the intracellular pathogen *Salmonella enterica* serovar Typhimurium initiates an anti-inflammatory transcriptional response in macrophages through its effector protein SteE. However, the target(s) and molecular mechanism of SteE remain unknown. Here, we demonstrate that SteE converts both the amino acid and substrate specificity of the host pleiotropic serine/threonine kinase GSK3. SteE itself is a substrate of GSK3, and phosphorylation of SteE is required for its activity. Remarkably, phosphorylated SteE then forces GSK3 to phosphorylate the non-canonical substrate signal transducer and activator of transcription 3 (STAT3) on tyrosine-705. This results in STAT3 activation, which along with GSK3 is required for SteE-mediated upregulation of the anti-inflammatory M2 macrophage marker interleukin-4Rα (IL-4Rα). Overall, the conversion of GSK3 to a tyrosine-directed kinase represents a tightly regulated event that enables a bacterial virulence protein to reprogram innate immune signaling and establish an anti-inflammatory environment.

## Highlights

• *Salmonella* effector SteE drives M2 macrophage polarization via GSK3 and STAT3
• SteE alters the substrate and amino acid specificity of the host kinase GSK3
• SteE enables GSK3, a S/T kinase, to phosphorylate STAT3 on Y705
• SteE requires GSK3-mediated phosphorylation to function

## Graphical Abstract

**Figure:**
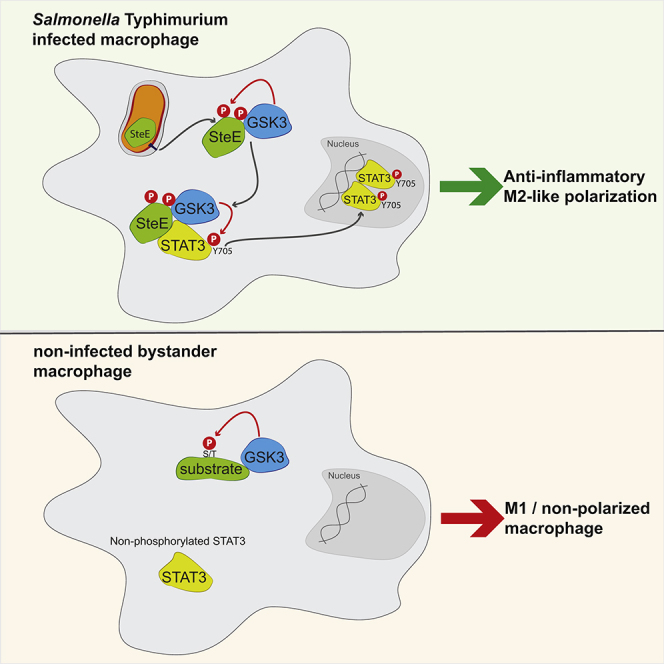

## Teaser

Numerous bacterial effectors modulate host immune responses via diverse biochemical activities. Panagi et al. report that the *Salmonella* effector SteE enables the pleiotropic host serine/threonine kinase GSK3 to phosphorylate a tyrosine residue on the non-canonical substrate STAT3. By triggering this substrate switch in GSK3, SteE creates an anti-inflammatory, infection-permissive environment.

## Introduction

The delivery of effector proteins into host cells enables pathogenesis of *Salmonella enterica* serovar Typhimurium. Effector translocation from intracellular bacteria is dependent on the *Salmonella* pathogenicity island-2 type III secretion system (T3SS) (Jennings et al., 2017). Numerous effectors suppress host inflammatory immune responses via diverse biochemical activities, including proteolysis (Jennings et al., 2018, Sun et al., 2016), arginine-GlcNAcylation (Gunster et al., 2017, Li et al., 2013), ubiquitination (Haraga and Miller, 2003), and eliminylation (Mazurkiewicz et al., 2008). As well as dampening host immune signaling pathways, it is now appreciated that *Salmonella* also induces anti-inflammatory pathways within the host. SteE (also referred to as STM2585 or SarA) stimulates the production of a key anti-inflammatory cytokine, interleukin-10 (IL-10), by activating the host transcription factor signal transducer and activator of transcription 3 (STAT3) (Jaslow et al., 2018). STAT3 is involved in many aspects of cell biology. After stimulation with cytokines such as IL-6 and IL-10, cytoplasmic STAT3 becomes phosphorylated on Y705 (Darnell et al., 1994, Schindler and Darnell, 1995). This results in STAT3 homodimerization, nuclear translocation, and expression of anti-inflammatory genes. It is known that *Salmonella* activates STAT3 in macrophages (Lin and Bost, 2004), but only recently was SteE identified as the key effector responsible (Jaslow et al., 2018). Although SteE interacts with STAT3, the mechanism driving STAT3 activation remains unknown.

More recently, it has been reported that SteE also directs macrophage polarization toward an anti-inflammatory M2-like state (Stapels et al., 2018). Macrophages are professional mononuclear phagocytes whose physiological state is plastic and context dependent. A simplified representation consists of classically activated pro-inflammatory M1 macrophages and alternatively activated M2 subtypes that are considered to be anti-inflammatory (Shapouri-Moghaddam et al., 2018). The polarization of macrophages to an M1 phenotype after stimulation with molecules such as lipopolysaccharide (LPS) and interferon-γ (IFN-γ) requires activation of downstream transcriptional regulators such as nuclear factor κB (NF-κB) and STAT1 (Shuai et al., 1994). The resulting macrophages are anti-microbial with high levels of nitric oxide (NO) and produce pro-inflammatory cytokines such as tumor necrosis factor α (TNF-α). In contrast, stimulation of macrophages with IL-4 or IL-10 leads to M2 polarization dependent on the activation of STAT3 or STAT6 (Wang et al., 2014). Intriguingly, emerging evidence suggests that M2-polarized macrophages are associated with intracellular *Salmonella* growth and persistence (Eisele et al., 2013, McCoy et al., 2012, Saliba et al., 2016). Additionally, studies utilizing murine models of salmonellosis have demonstrated that SteE is important for the virulence and long-term persistence of *Salmonella* at systemic sites of infection (Jaslow et al., 2018, Lawley et al., 2006, Niemann et al., 2011). Despite this progress, the molecular details of how SteE drives M2-like polarization are lacking entirely, and the link between SteE-induced STAT3 activation and macrophage polarization is unknown. It is also unclear how SteE functions biochemically, because it is a small and apparently non-enzymatic protein. Here, we report that SteE alters the substrate specificity of host glycogen synthase kinase 3 (GSK3) and thus endows this serine/threonine (S/T) kinase with the ability to phosphorylate a tyrosine residue on the non-canonical substrate STAT3, ultimately driving macrophage polarization.

## Results

### *Salmonella*-Mediated M2 Macrophage Polarization Is SteE and STAT3 Dependent

**Figure 1:**
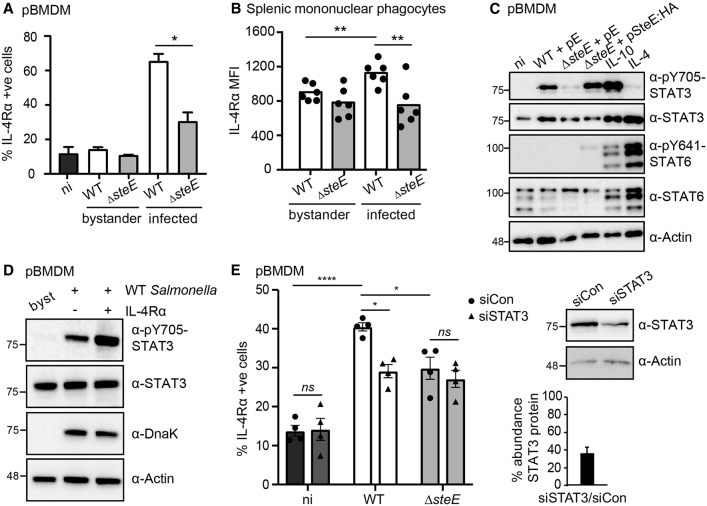
M2 Macrophage Polarization Is SteE and STAT3 Dependent (A) Percentage of IL-4Rα^+^ pBMDMs in naive, non-infected bystander or infected cells 17 h after uptake. Cells were infected with WT or *steE* mutant *Salmonella* carrying the fluorescent plasmid pFCcGi (see Figure S1A for gating). Data represent the mean and SEM of four independent experiments (one-way ANOVA with Dunnett’s multiple-comparison test, ^∗^p < 0.05). (B) Median fluorescent intensity (MFI) of IL-4Rα signal from CD11b^+^MHCII^+^F4/80^+^ and Ly6G^−^ mononuclear phagocytes isolated from the spleens of C57BL/6 *Nramp*^+/+^ mice (see Figure S1B for gating). Mice were infected with WT or *steE* mutant *Salmonella* via intraperitoneal inoculation, and spleens were harvested 10 days after infection. Bars represent the geometric median; dots represent individual mice. Significance was calculated with a two-tailed Mann-Whitney test (^∗^p < 0.05, ^∗∗^p < 0.01). Data represent three independent experiments with four to six mice analyzed per group per experiment. (C) Protein immunoblots of whole-cell lysates derived from primary bone-marrow-derived macrophages (pBMDMs) infected with WT, *steE* mutant, or *steE* mutant *Salmonella* carrying a plasmid expressing SteE:HA. pE denotes that the strain carries an empty plasmid. Lysates were harvested 17 h after uptake. Alternatively, pBMDMs were stimulated with 20 mg/mL IL-4 or IL-10 for 17 h, as indicated. Immunoblots are representative of three independent experiments. (D) pBMDMs were infected with WT *Salmonella* carrying the fluorescent plasmid pFCcGi for 18 h and then FACS sorted according to whether they were non-infected bystanders (bysts), infected IL4Rα^−^, or infected IL4Rα^+^. Only infected cells with a similar bacterial burden were collected (Figure S1C). Sorted cells were then lysed and analyzed by immunoblot with the indicated antibodies. Data are representative of two independent repeats. (E) Percentage of IL-4Rα^+^ non-infected or infected pBMDMs 17 h after uptake of WT + pFCcGi or *steE* mutant + pFCcGi *Salmonella* after treatment with control (siCon) or STAT3 siRNA for 2 days. Data represent mean and SEM of four independent experiments (two-way ANOVA with Tukey’s multiple-comparison test, ^∗^p < 0.05, ^∗∗^p < 0.01; ns, not significant). Inset: immunoblot for STAT3 levels in control or STAT3 siRNA-treated pBMDMs with quantification from three independent repeats shown as mean and SEM.

Infection of primary bone-marrow-derived macrophages (pBMDMs) by *Salmonella* Typhimurium polarizes cells into an anti-inflammatory M2-like state that is dependent on SteE (Stapels et al., 2018). In agreement, we found an SteE-dependent upregulation of the M2 marker IL-4Rα in infected, but not non-infected, bystander cells in both pBMDMs (Figures 1A and S1A) and splenic mononuclear phagocytes (Figures 1B and S1B). This shows that SteE-dependent macrophage polarization is cell intrinsic, even when other signaling events and immune cells are present. M2 polarization is associated with activated STAT3 (pY705) and STAT6 (pY641) (Wang et al., 2014), and in agreement with others (Jaslow et al., 2018), SteE induced STAT3 phosphorylation (Figure 1C). However, infection with wild-type (WT) *Salmonella* did not induce STAT6 phosphorylation (Figure 1C). Therefore, we hypothesized that SteE mediates the polarization of macrophages through phosphorylation and activation of STAT3.

To test this, we analyzed the relative degree of STAT3 phosphorylation in IL-4Rα^−^ and IL-4Rα^+^ cell populations. pBMDMs were infected with fluorescent WT *Salmonella* (Figueira et al., 2013) and sorted into three populations by fluorescence-activated cell sorting (FACS): non-infected bystanders, infected IL-4Rα^−^, and infected IL-4Rα^+^. The gating strategy selected an equivalent bacterial burden in the different infected populations (Figure S1C). Virtually no pY705-STAT3 was detected in cell lysates from non-infected bystanders. In contrast, STAT3 phosphorylation was detected in infected samples, which showed a stronger pY705-STAT3 signal in infected IL-4Rα^+^ cells than in IL-4Rα^−^ cells (Figure 1D). To test directly whether STAT3 is required for *Salmonella*-mediated upregulation of IL-4Rα protein levels, we treated pBMDMs with a small interfering RNA (siRNA)-targeting STAT3 and then infected cells with fluorescent WT or *steE* mutant *Salmonella*. In control siRNA-treated cells, WT *Salmonella* caused an SteE-dependent upregulation in IL-4Rα levels. In contrast, WT *Salmonella* did not increase the levels of IL-4Rα significantly in siSTAT3-treated pBMDMs (Figures 1E and S1D). We conclude that SteE drives cell-intrinsic M2-like macrophage polarization through the activation of the transcription factor STAT3.

### SteE Interacts with GSK3α and GSK3β as well as STAT3

**Figure 2:**
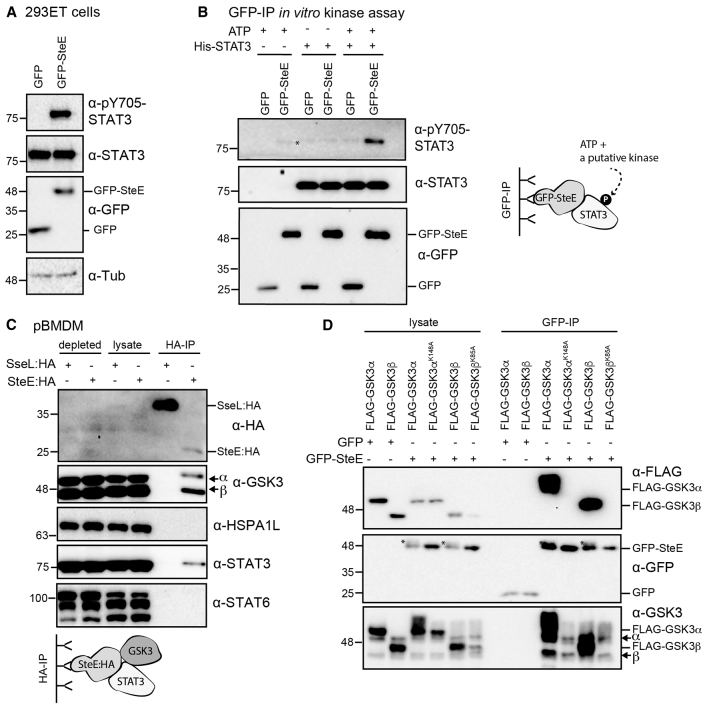
SteE Interacts with Catalytically Active GSK3 and STAT3 (A) Whole-cell lysates from 293ET cells expressing GFP or GFP-SteE were analyzed by immunoblot with antibodies against STAT3, pY705-STAT3, GFP, and tubulin (Tub) as a loading control. Data are representative of three independent experiments. (B) GFP or GFP-SteE was expressed in 293ET cells, immunoprecipitated, and assessed for its ability to phosphorylate exogenously added recombinant His-STAT3 in an *in vitro* kinase assay. The ^∗^ represents endogenous phosphorylated STAT3 that was detected upon immunoprecipitation with GFP-SteE but not with GFP. Data are representative of three independent repeats. (C) pBMDMs were infected with *steE* mutant *Salmonella* carrying pWSK29-SteE:HA or *sseL* mutant *Salmonella* carrying pWSK29-SseL:HA. 17 h after uptake, HA-tagged effectors were immunoprecipitated from cell lysates and assessed for their ability to bind endogenous GSK3, STAT3, STAT6, or HSPA1L as indicated. Immunoblots are representative of three independent experiments. (D) GFP or GFP-SteE, expressed in 293ET cells, was immunoprecipitated and assessed for its ability to interact with the indicated co-expressed FLAG-tagged GSK3 variants and endogenous GSK3 (indicated with arrows). Data are representative of three experiments. The ^∗^ indicates a higher-molecular-weight form of SteE.

Next, we investigated how SteE induces STAT3 phosphorylation and activation. Exploiting the fact that GFP-tagged SteE is sufficient to induce STAT3 phosphorylation in HeLa cells (Jaslow et al., 2018; Figure S2A) and 293ET cells (Figure 2A), we tested whether GFP-SteE immunoprecipitated from 293ET cells could phosphorylate recombinant His-STAT3 in an *in vitro* kinase assay. Immunoprecipitated GFP-SteE induced phosphorylation of His-STAT3 upon the addition of ATP, whereas the GFP control sample did not (Figure 2B). This suggests that either SteE is a kinase or it associates with a kinase to which it remains bound during immunoprecipitation.

Because SteE is composed of only 157 amino acids and does not resemble either a bacterial or mammalian kinase, we hypothesized that a host interaction partner (or partners) of SteE might be responsible for STAT3 phosphorylation. To this end, GFP or GFP-SteE was immunoprecipitated from 293ET cells, and the samples were analyzed by mass spectrometry. The identified proteins that were unique or highly enriched in the GFP-SteE samples from three independent repeats are shown in Table 1. We then tested whether SteE:HA, translocated by *Salmonella*, interacted with the top three hits: heat shock 70 kDa protein 1-like (HSPA1L), GSK3α, and GSK3β. HSPA1L was not detected in SteE:HA immunoprecipitated samples from infected pBMDM cells (Figure 2C). In contrast, translocated SteE:HA, but not the unrelated effector protein SseL (SseL:HA), immunoprecipitated endogenous GSK3α and GSK3β from pBMDMs (Figure 2C), 293ET cells (Figure S2B), and HeLa cells (Figure S2C). Exogenously expressed SteE has previously been found to interact with STAT3 (Jaslow et al., 2018), and translocated SteE:HA, but not SseL:HA, also interacted with endogenous STAT3 (Figure 2C). No interaction was detected between STAT6 and SteE:HA (Figure 2C). The activated Y705-phosphorylated form of STAT3 was also detected in SteE:HA immunoprecipitated samples, providing additional evidence that STAT3 is phosphorylated in an SteE-containing complex (Figure S2B). Therefore, GSK3α, GSK3β, and STAT3 interact with SteE in infected cells.

**Table 1:** Putative SteE Interaction Partners Identified by Mass Spectrometry

Protein Name	Gene Name	Unique Peptides	Average Ion Score	Specific/Non-specific Ratio
Heat shock 70 kDa protein 1-like	*HSPA1L*	22/26/23	36,891 (17,867)	∞
Glycogen synthase kinase-3 alpha	*GSK3α*	19/15/20	4,814 (949)	∞
Glycogen synthase kinase-3 beta	*GSK3β*	15/14/18	4,741 (356)	∞
Heat shock protein 105 kDa	*HSPH1*	18/24/26	2,380 (709)	∞
Heat shock 70 kDa protein 4L	*HSPA4L*	12/8/9	634 (177)	∞
GrpE protein homolog 1, mitochondrial	*GRPEL1*	6/11/8	914 (495)	∞
BAG family molecular chaperone regulator 5	*BAG5*	4/4/6	442 (208)	∞
WD repeat-containing protein 54	*WDR54*	1/1/1	136 (44)	∞
Heat shock 70 kDa protein 1A	*HSPA1A*	51/60/49	92,417 (32,920)	43,057 (3,755)
Heat shock cognate 71 kDa protein	*HSPA8*	38/46/38	17,707 (3,301)	6,824 (843)
78 kDa glucose-regulated protein	*HSPA5*	33/41/22	7,252 (2,344)	4,819 (485)
Stress-70 protein, mitochondrial	*HSPA9*	34/45/36	8,546 (3,769)	2,416 (489)
Heat shock 70 kDa protein 4	*HSPA4*	34/35/34	3,868 (384)	1,440 (114)
E3 ubiquitin-protein ligase CHIP	*STUB1*	11/12/9	1,679 (184)	1,192 (56)
BAG family molecular chaperone regulator 2	*BAG2*	4/6/6	611 (236)	491 (33)

Proteins that were specific or highly enriched in the GFP-SteE sample, compared with the GFP control, are shown. Data were obtained from three independent experiments, and the number of unique peptides from each experiment shown together with an average ion score; SD is given in parentheses.

Through the analysis of GSK3β deletion mutants, the minimal region required for the interaction with SteE was identified as amino acids 56–384; truncations into the kinase domain from either the N or C terminus resulted in loss of the interaction (Figure S2D). This led us to hypothesize that GSK3 kinase activity is required for the interaction between GSK3 and SteE. FLAG-tagged GSK3α or GSK3β interacted specifically with co-expressed immunoprecipitated GFP-SteE, whereas the catalytically inactive ATP-binding-deficient point mutants (GSK3α^K148A^ and GSK3β^K85A^) did not (Figure 2D). Endogenous GSK3 was efficiently immunoprecipitated from all GFP-SteE-containing samples (Figure 2D). This shows that SteE only interacts with the enzymatically active form of GSK3.

### Catalytically Active GSK3 Is Required for SteE-Induced STAT3 Activation and Macrophage Polarization

**Figure 3:**
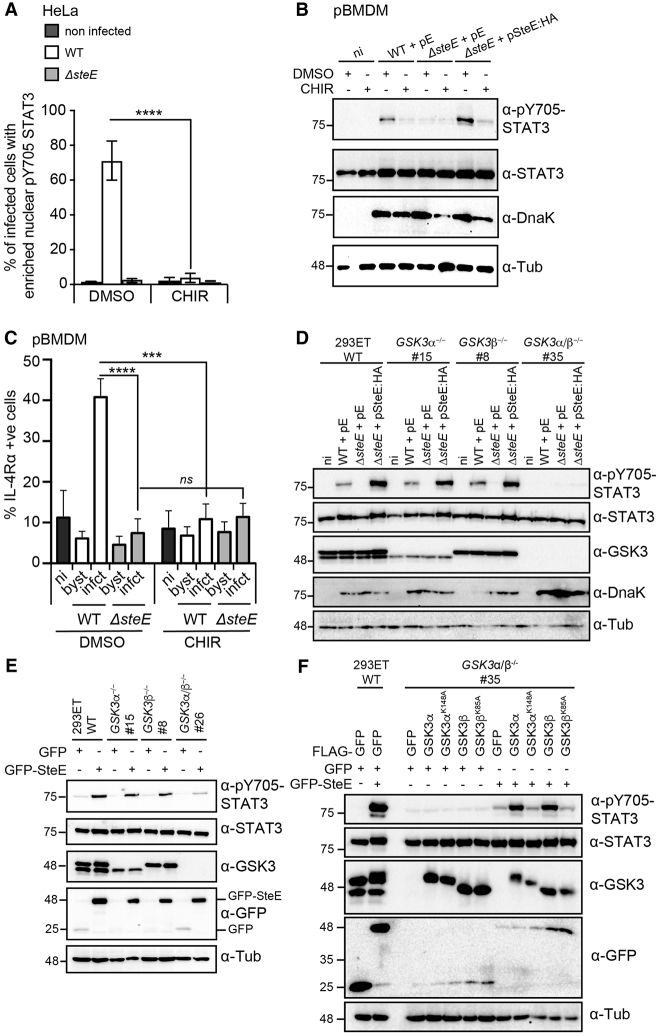
GSK3 Is Required for *Salmonella*-Induced Phosphorylation of STAT3 and Macrophage Polarization (A) HeLa cells were infected with the indicated *Salmonella* strains and then treated with either DMSO or 5 μM GSK3 inhibitor CHIR99021 1 h after infection. 17 h after infection, the cells were fixed, permeabilized, and then labeled for Y705-phosphorylated STAT3, CSA1 (*Salmonella*), and DAPI (nucleus) (Figure S3A). The frequency of infected cells with enriched nuclear pY705-STAT3 signal was enumerated by eye. Data are the mean and SEM of three independent repeats where at least 100 infected cells were blind scored. ^∗∗∗∗^p < 0.0001, one-way ANOVA with Dunnett’s post hoc analysis for multiple comparisons. (B) pBMDMs were infected with the indicated *Salmonella* strains for 17 h and treated with DMSO or 5 μM CHIR99021 1 h after uptake. Cell lysates were analyzed by immunoblotting with antibodies against active STAT3 (pY705), STAT3, DnaK for a *Salmonella* infection control, or tubulin (Tub) as a loading control. Data represent the findings of three independent experiments. pE, empty vector. (C) Percentage of IL-4Rα^+^ pBMDMs in non-infected (ni), bystander (byst), or infected (infct) cells with a restricted growth gate (Figure S3D) 17 h after uptake of WT or *steE* mutant *Salmonella* carrying the fluorescent plasmid pFCcGi. Where indicated, cells were treated with 5 μM CHIR99021 1 h after uptake. Data represent mean and SEM of three independent experiments (two-way ANOVA with Tukey’s multiple-comparison test, ^∗∗∗^p < 0.001, ^∗∗∗∗^p < 0.0001; ns, not significant). (D) WT or CRISPR/Cas9-generated *GSK3α*^−/−^, *GSK3β*^−/−^, or *GSK3α/β*^−/−^ 293ET cells were infected with the indicated *Salmonella* strains for 17 h before whole-cell lysates were immunoblotted with the indicated antibodies. The # indicates the clone used, and pE denotes empty vector. Data are representative of three independent experiments. (E) GFP or GFP-SteE was expressed in either WT 293ET cells or the indicated GSK3 knockout cell lines, and cell lysates were immunoblotted with the indicated antibodies. Immunoblots are representative of three independent experiments. (F) GFP or GFP-SteE was co-expressed with the indicated FLAG-tagged GSK3 active or inactive variants in either WT 293ET cells or *GSK3α/β*^−/−^ 293ET cells. Cell lysates were then analyzed by immunoblot for the activation of STAT3. Data are representative of three independent experiments.

GSK3α and GSK3β are S/T kinases that share a highly similar (98%) kinase domain and phosphorylate several common substrates but are not redundant. GSK3α negatively regulates STAT3 activity in atherosclerosis (McAlpine et al., 2015), whereas GSK3β indirectly promotes the phosphorylation of Y705 on STAT3 via membrane-associated tyrosine kinases (Beurel and Jope, 2008, Gao et al., 2017). To investigate whether GSK3 activity is required for *Salmonella*-mediated phosphorylation of STAT3, we used immunofluorescence microscopy to monitor pY705-STAT3 localization in *Salmonella*-infected HeLa cells treated with the GSK3 kinase inhibitor CHIR99021. As expected, infection of DMSO-treated cells with WT *Salmonella* resulted in a strong nuclear accumulation of pY705-STAT3 (Figures 3A and S3A). In contrast, very few cells showed nuclear accumulation of pY705-STAT3 in *steE-*mutant-infected or CHIR99021-treated cells, suggesting that nuclear accumulation of pY705-STAT3 is dependent on both SteE and GSK3 activity (Figure 3A). Next, the ability of SteE to induce Y705-STAT3 phosphorylation was examined by immunoblotting. In both pBMDMs and HeLa cells, WT-*Salmonella*-induced Y705-STAT3 phosphorylation was strongly inhibited by the addition of CHIR99021 (Figures 3B and S3B). CHIR99021 did not reduce residual Y705-phosphorylated STAT3 in *steE* mutant infected cells (Figures 3B and S3B), IL-10-induced STAT3 activation, or the activation of STAT6 by IL-4 or IL-10 (Figure S3C). This suggests that CHIR99021 specifically inhibits SteE-dependent STAT3 phosphorylation. Finally, we tested whether CHIR99021 also prevented SteE-mediated upregulation of IL-4Rα in pBMDMs. CHIR99021 caused a minor reduction in DnaK levels in pBMDMs (Figure 3B) but not in HeLa cells (Figure S3B). This decrease in bacterial burden was quantified in pBMDMs by flow cytometry (Figures S3D and S3E), and a refined gate that selected for a similar bacterial burden in both DMSO- and CHIR99021-treated samples was used (Figures S3D and S3F). With this gate, *Salmonella* infection caused ∼40% of infected cells to become IL-4Rα^+^ in DMSO-treated cells but only ∼10% of infected cells to become IL-4Rα^+^ in the CHIR99021-treated cells (Figure 3C). CHIR99021 did not significantly alter the amount of IL-4Rα^+^ cells infected with *steE*-mutant bacteria (Figure 3C) or when macrophages were polarized with IL-10 (Figure S3G). Collectively, these data show that GSK3 kinase activity is required specifically for SteE- but not IL-10-mediated Y705-STAT3 phosphorylation and upregulation of the M2 marker IL-4Rα during *Salmonella* infection.

Given that SteE interacted with both GSK3α and GSK3β (Figure 2C), we next tested whether either one or both kinases are required for SteE-induced STAT3 activation. To this end, *GSK3α*^−/−^, *GSK3β*^−/−^, and *GSK3α/β*^−/−^ knockout 293ET cell lines were generated with the use of CRISPR/Cas9. Infection of WT, *GSK3α*^−/−^, or *GSK3β*^−/−^ cells with WT *Salmonella* resulted in a similar amount of Y705-phosphorylated STAT3, which was no longer detected in lysates from *GSK3α/β*^−/−^ cells, despite similar total STAT3 levels (Figure 3D). *steE* mutant bacteria carrying a plasmid expressing SteE:HA restored STAT3 Y705 phosphorylation in infected WT, *GSK3α*^−/−^, and *GSK3β*^−/−^ cells but not in *GSK3α/β*^−/−^ cells (Figure 3D). This shows that in the context of *Salmonella*-induced STAT3 activation, GSK3α and GSK3β are redundant.

During the course of these experiments, we found that although similar amounts of effector were detected inside bacteria (pellet fraction), translocated SteE:HA (post-nuclear supernatant [PNS] fraction) was barely detected in the absence of GSK3 (Figure S4A). This requirement for GSK3 was specific to SteE; translocated SseL:HA was detected in CHIR99021-treated and in *GSK3α/β*^−/−^ cells (Figure S4A), and SseL:HA and another effector PipB:HA were detected by immunofluorescence microscopy in DMSO- and CHIR99021-treated WT 293ET cells and *GSK3α/β*^−/−^ cells (Figure S4B). We conclude that GSK3 prevents degradation of translocated SteE.

In contrast, exogenously expressed GFP-SteE remained stable in the absence of GSK3 (Figure 3E). Therefore, to test the requirement of GSK3 in STAT3 phosphorylation, independently of its requirement in preventing SteE degradation, we expressed GFP or GFP-SteE in GSK3-knockout cells. In WT, *GSK3α*^−/−^, or *GSK3β*^−/−^ cells, GFP-SteE induced a similar amount of STAT3 Y705 phosphorylation without altering the levels of total STAT3. In contrast, in three independently generated *GSK3α/β*^−/−^ clones, GFP-SteE induced minimal STAT3 Y705 phosphorylation (Figures 3E and S4C). Finally, we analyzed pY705-STAT3 levels from *GSK3α/β*^−/−^ cells expressing either active or inactive GSK3 variants. Only the expression of WT GSK3α or WT GSK3β was sufficient to enable GFP-SteE-mediated STAT3 activation (Figure 3F). In summary, the kinase activity of either GSK3α or GSK3β is required for both stabilizing translocated SteE and enabling SteE-mediated Y705-STAT3 phosphorylation and thereby macrophage polarization.

### SteE Enables GSK3 to Form a Complex with STAT3 in which SteE and STAT3 Are Phosphorylated

**Figure 4:**
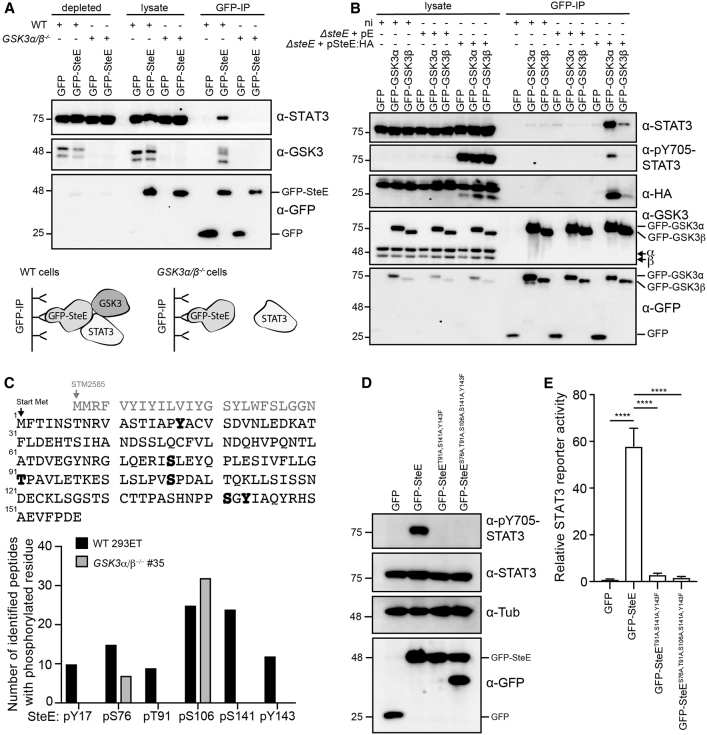
SteE Enables GSK3 to Form a Complex with STAT3 in which SteE and STAT3 Are Phosphorylated (A) WT or *GSK3α/β*^−/−^ 293ET cells expressing GFP or GFP-SteE were lysed, subjected to a GFP immunoprecipitation, and assessed for binding to endogenous STAT3 and GSK3 by immunoblot. Data are representative of three independent repeats. (B) 293ET cells stably expressing GFP, GFP-GSK3α, or GFP-GSK3β were left non-infected (ni) or infected with *steE* mutant *Salmonella* carrying either empty plasmid (pE) or pWSK29-SteE:HA. 17 h after uptake, cell lysates were subject to GFP immunoprecipitation (IP) and lysate, and IP samples were analyzed by immunoblotting for HA, endogenous STAT3, activated STAT3 (pY705), endogenous GSK3, and GFP. Data represent the findings of three independent experiments. (C) The amino acid sequence and numbering of SteE are shown in black. Amino acids identified as phosphorylated are shown in bold. The number of peptides with each phosphorylated residue, as detected by mass spectrometry, is shown for GFP-SteE expressed in either WT or *GSK3α/β*^−/−^ 293ET cells. See Figure S5B for protein expression. The data are obtained from three independent repeats. (D) Whole-cell lysates from 293ET cells transiently expressing GFP, GFP-SteE, or the indicated GFP-tagged SteE mutants were analyzed by immunoblot with antibodies against STAT3, pY705-STAT3, tubulin (Tub), and GFP. Data are representative of three independent experiments. (E) Luciferase activity in cell lysates from 293ET cells co-transfected with plasmids encoding a STAT3-dependent *Firefly* luciferase, a constitutively expressed *Renilla* luciferase, and GFP or the indicated GFP-SteE variant. Data are presented as the fold change in STAT3 reporter activity from GFP-expressing cells and represent the mean and SEM of four independent experiments. Statistical significances were calculated from WT GFP-SteE. ^∗∗∗∗^p < 0.0001, one-way ANOVA with Dunnett’s post hoc analysis for multiple comparisons.

Next, we investigated the molecular basis for the requirement of GSK3 in SteE-induced STAT3 phosphorylation. Because STAT3 becomes phosphorylated in an SteE-containing complex (Figure 2B), we first questioned whether GSK3 is required for the interaction between SteE and STAT3. Exogenously expressed GFP-SteE, which was readily detected in both WT and *GSK3α/β*^−/−^ cells, interacted with endogenous STAT3 in WT 293ET cells but not in *GSK3α/β*^−/−^ cells (Figure 4A), showing that GSK3 is critical for the interaction of SteE with STAT3.

Because GSK3 can interact with STAT3 (Beurel and Jope, 2008), we tested whether this occurs with or without SteE. In non-infected 293ET cells, or in cells infected with *steE* mutant bacteria, an interaction between GFP-GSK3α or GFP-GSK3β and STAT3 was not detected. However, upon infection with *steE* mutant bacteria expressing SteE:HA, both GFP-GSK3α and GFP-GSK3β interacted with STAT3 (Figure 4B). In the presence of SteE, Y705-phosphorylated STAT3 was also detected in the GFP-GSK3α immunoprecipitated samples (Figure 4B). To investigate this further, we carried out *in vitro* kinase assays after immunoprecipitation of GSK3. Recombinant GST-STAT3 was phosphorylated only when GFP-GSK3α or GFP-GSK3β was immunoprecipitated from cells infected with bacteria expressing SteE:HA. This was enhanced by addition of ATP (Figure S5A). We conclude that SteE potentiates a GSK3-STAT3 interaction that results in STAT3 phosphorylation.

After analysis of our GSK3-immunoprecipitated *in vitro* kinase assays, we noted several higher-molecular-weight bands on the anti-HA immunoblot for detection of SteE:HA (Figure S5A). In addition, bacterially delivered SteE:HA migrated as a doublet (red arrows in Figures S2B and S4A) and transfected GFP-SteE migrated as a doublet in cells expressing active GSK3α or GSK3β (Figure 2D). Together, these findings imply a post-translational SteE modification that would be consistent with phosphorylation. To identify the putative phosphorylated amino acids of SteE, we expressed GFP-SteE in WT or *GSK3α/β*^−/−^ 293ET cells (Figure S5B), immunoprecipitated it, and analyzed it by mass spectrometry. The start site of SteE (STM2585) is misannotated (Baek et al., 2017), resulting in an additional 24 amino acids (Figure 4C). We therefore used the shorter SteE version that has a consensus Shine-Dalgarno sequence directly upstream of the AUG codon (Figure S5C), and when this version is HA tagged on the C terminus, it yields a protein of identical molecular mass to the “long” form of SteE (Figure S5C). Phosphorylation at S76 and S106 was detected in GFP-SteE expressed in either WT or *GSK3α/β*^−/−^ cells, whereas phosphorylation at Y17, T91, S141, and Y143 was detected only in GFP-SteE isolated from WT cells (Figure 4C).

To assess the importance of SteE phosphorylation in STAT3 activation, we muted single S/T residues to alanine, whereas Y143 was mutated to phenylalanine (S76A, T91A, S106A, S141A, and Y143F). All mutants were similarly expressed, and each induced equivalent or better STAT3 luciferase-reporter activation when compared with GFP-SteE (Figure S5D). However, GFP-SteE^T91A/S141A/Y143F^, mutated at three of the putative GSK3 phosphorylation sites, was unable to induce Y705-STAT3 phosphorylation (Figure 4D), did not activate the STAT3 luciferase reporter (Figure 4E), and was as inactive as GFP-SteE^S76A/T91A/S106A/S141A/Y143F^ (Figures 4D and 4E). Together, our data show that GSK3 is required for the interaction between SteE and STAT3 and that GSK3-dependent phosphorylation of SteE is critical for SteE-induced STAT3 activation.

### SteE Converts GSK3 to a Tyrosine Kinase with Substrate Specificity for STAT3

Because GSK3α and GSK3β are S/T kinases and STAT3 becomes phosphorylated on Y705, we first hypothesized that a tyrosine kinase is required. To test this, we screened an array of tyrosine-kinase inhibitors during infection of cells with *steE*-mutant *Salmonella* expressing SteE:HA and monitored STAT3 phosphorylation. Only the addition of the GSK3 inhibitor CHIR99021 suppressed STAT3 phosphorylation (Figure S6A). Canonical cytokine-mediated STAT3 phosphorylation is mediated by JAK proteins (Darnell et al., 1994). However, because STAT3 phosphorylation by *Salmonella* is independent of IL-6 and IL-10 (Lin and Bost, 2004), it was not surprising that the addition of the JAK inhibitors tofacatinib and cerdulatinib did not prevent *Salmonella*-induced STAT3 phosphorylation (Figure S6A). These data suggest that STAT3 becomes phosphorylated by a non-canonical mechanism.

Next, we tested whether SteE promotes the recruitment of additional proteins required for STAT3 phosphorylation to the GSK3 complex. To this end, His-MBP-SteEΔN20, or His-MBP as a negative control, was expressed in *E. coli* and purified (Figure S6B). We removed the first 20 amino acids, which are not required for function (Figure S6C), to improve solubility. GFP-GSK3α or GFP-GSK3β, immunoprecipitated from non-infected 293ET cells, resulted in STAT3 Y705 phosphorylation only when recombinant His-MBP-SteEΔN20 and ATP were added but not when His-MBP was added (Figure S6D). Notably, His-MBP-SteEΔN20 and STAT3 in the GFP sample did not yield STAT3 phosphorylation, highlighting the essential role of GSK3 in this process. The finding that recombinant SteE can replace infection- or transfection-delivered SteE shows that the proteins required for STAT3 phosphorylation are already present in the GSK3 complex.

**Figure 5:**
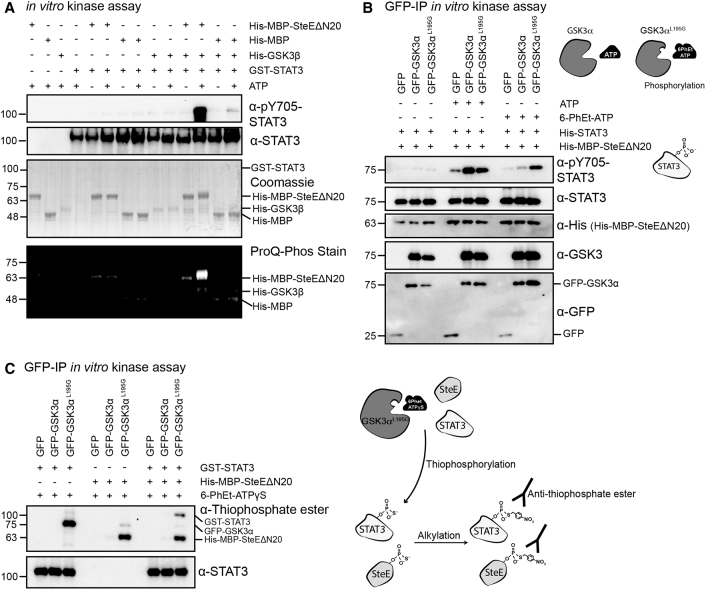
SteE Enables GSK3 to Phosphorylate STAT3 (A) *In vitro* kinase assays containing 5 μg recombinant His-GSK3β, 12.5 μg His-MBP or His-MBP-SteEΔN20, and 0.4 μg GST-STAT3, all with or without 1 mM ATP, were assayed by immunoblot, Coomassie stain, and Pro-Q Diamond phosphoprotein stain. Data are representative of three repeats. (B) GFP, WT GFP-GSK3α, and GFP-GSK3α^L195G^ were expressed in 293ET cells, immunoprecipitated, and assessed by immunoblot for their ability to phosphorylate exogenously added recombinant His-STAT3 in an *in vitro* kinase assay containing His-MBP-SteEΔN20 and either no ATP, ATP, or 6-PhEt-ATP. Data are representative of three experiments. (C) *In vitro* kinase assays containing immunoprecipitated GFP, WT GFP-GSK3α or GFP-GSK3α^L195G^; and 2 μg GST-STAT3 and/or 1.6 μg His-MBP-SteEΔN20, as indicated were incubated with N6-PhEt-ATPγS as the phosphate donor. Samples were assayed by immunoblot with the indicated antibodies. Data are representative of two experiments.

Despite exhibiting only serine and threonine kinase activity toward exogenous substrates, GSK3 displays tyrosine-directed auto-phosphorylation, which results in phosphorylation of Y279 (GSK3α) and Y216 (GSK3β) (Cole et al., 2004). We therefore hypothesized that GSK3, when complexed to SteE, directly phosphorylates STAT3 on Y705. We expressed His-GSK3β in insect cells, purified the protein, and carried out *in vitro* kinase assays in the presence of recombinant His-MBP-SteEΔN20. Using Pro-Q Diamond phosphoprotein gel stain, we detected phosphorylation of His-MBP-SteEΔN20 but not of His-MBP in the GSK3β + ATP sample, providing strong evidence that SteE is a direct substrate of GSK3 (Figure 5A). Analysis of STAT3 activation revealed that Y705-phosphorylated STAT3 was detected only when recombinant His-GSK3β, His-MBP-SteEΔN20, and GST-STAT3 were incubated in the presence of ATP (Figure 5A). Mass spectrometry confirmed that in the presence of recombinant GSK3β and SteE, STAT3 became phosphorylated on Y705 (Figure S6E). The percentage of detected phosphorylated to non-phosphorylated peptides was analyzed for peptides containing the phosphorylated STAT3 residues Y705 or S727. Whereas the addition of GSK3β to the His-MBP-SteEΔ20 + GST-STAT3 sample did not alter the percentage of phosphorylated S727-STAT3 peptides, it significantly increased the percentage of pY705 peptides from 17% to 56% (Figure S6F). Because GSK3 purified with two different affinity tags (GFP and His) from two different cell types (human and insect) can still result in STAT3 phosphorylation upon the addition of SteE, it strongly suggests that the GSK3-SteE complex phosphorylates STAT3 at Y705.

To establish whether GSK3 is directly responsible for catalyzing the hydrolysis of the donating ATP in the context of SteE-mediated STAT3 phosphorylation, we mutated the ATP-binding pocket of GSK3α to accommodate a larger ATP analog (Hertz et al., 2010). The structurally conserved bulky leucine at the “gatekeeper” position in the kinase active site was mutated to a glycine (Chen et al., 2017). GSK3α^L195G^ phosphorylated STAT3 less efficiently than WT GSK3α when incubated with ATP (Figure 5B), which is consistent with a previous report (Chen et al., 2017). Importantly, GSK3α^L195G^ was able to use the bulky N6-PhEt-ATP, and STAT3 became phosphorylated at Y705, which we detected with the anti-pY705-STAT3 antibody. In contrast, WT GSK3 showed very poor STAT3 Y705 phosphorylation when incubated with the ATP analog (Figure 5B). This shows that GSK3α hydrolyses the phosphate group from ATP, providing strong evidence that GSK3 is the kinase responsible for STAT3 phosphorylation. To test whether STAT3 receives this phosphate, we replaced the bulky ATP analog with a bulky synthetic N6-PhEt-ATPγS analog to thiophosphorylate the protein substrates of GSK3. Alkylation of thiophosphorylated substrates generates a bio-orthogonal thiophosphate ester tag that can be detected by immunoblotting with a thiophosphate ester specific antibody. *In vitro* kinase assays with GFP-GSK3α^L195G^ led the anti-thiophosphate ester antibody to detect three proteins, corresponding in molecular mass to His-MBP-SteEΔN20 (63 kDa), GFP-GSK3α^L195G^ (75 kDa), and GST-STAT3 (120 kDa) (Figure 5C). In the absence of His-MBP-SteEΔN20, only a faint band was detected at the molecular mass corresponding to GST-STAT3. The increase in intensity upon the addition of His-MBP-SteEΔN20 demonstrates that SteE facilitates GSK3α^L195G^-mediated phosphorylation of STAT3. Phosphorylation of GST-STAT3 was not detected in the GFP-GSK3α sample, even in the presence of SteE, providing unequivocal evidence that GSK3 is the kinase responsible for ATP hydrolysis and STAT3 phosphorylation (Figure 5C).

**Figure 6:**
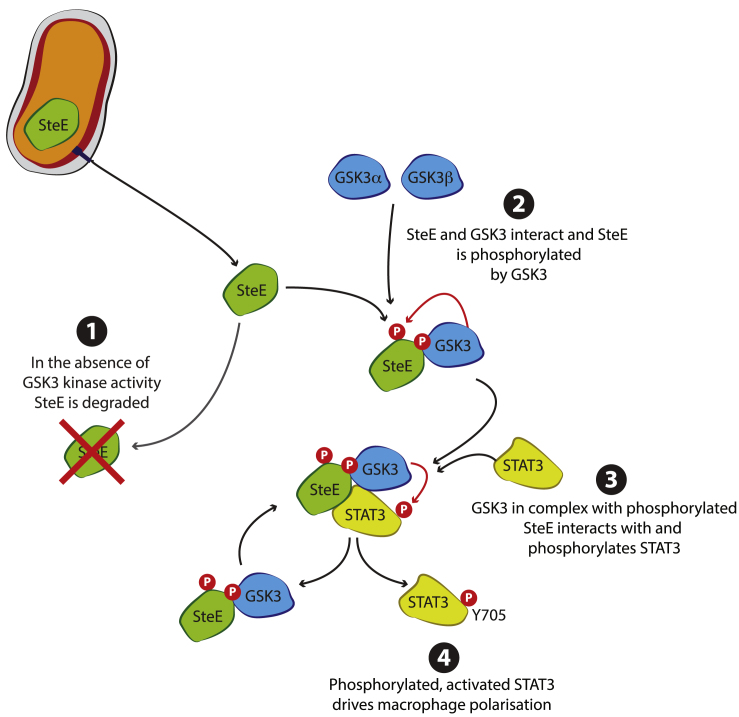
Model of SteE Action After translocation, SteE interacts with and is phosphorylated by GSK3. GSK3 in complex with phosphorylated SteE then interacts with and phosphorylates STAT3 on Y705. Activated STAT3 drives macrophage polarization.

We conclude that in the presence of SteE, the substrate and amino acid specificity of GSK3 is altered. SteE is phosphorylated by GSK3 on several residues, and this is required for SteE function. GSK3-mediated phosphorylation of SteE then permits the interaction with and phosphorylation of the non-canonical substrate STAT3 on Y705. Overall, the action of SteE culminates in an anti-inflammatory immune response within the infected cell (Figure 6).

## Discussion

Protein phosphorylation is a widespread reversible post-translational modification that regulates signal transduction. Here, we demonstrate that SteE-induced anti-inflammatory macrophage polarization requires phosphorylation of the host transcription factor STAT3 by the noncanonical kinase GSK3. According to current dogma, GSK3 displays strict S/T kinase activity toward exogenous substrates, and like other S/T kinases, it has a lysine residue two amino acids downstream of the proton acceptor. Dual-specificity kinases (DSKs) can phosphorylate both S/T and tyrosine residues on target proteins. GSK3 can phosphorylate its activation-loop tyrosine residue in *cis*, but because this event is exclusively intramolecular, GSK3 is not considered to be a “true” DSK. Our findings establish that in the context of *S*. *typhimurium* infection, GSK3, in complex with SteE, is converted from a S/T kinase to a DSK that *trans* phosphorylates STAT3 on Y705.

How might GSK3 phosphorylate Y705 on STAT3? Various mechanisms for autotyrosine phosphorylation among the CMGC group of kinases (of which GSK3 is a member) have been reported. For GSK3, autophosphorylation is thought to be a one-time event that depends on the chaperone HSP90 (Cole et al., 2004, Lochhead et al., 2006). In contrast, *Drosophila* dDyrk2 contains an N-terminal autophosphorylation accessory (NAPA) region that is required for tyrosine autophosphorylation (Kinstrie et al., 2010), whereas DYRK1A is thought to exist in a dynamic equilibrium between two conformational states, one capable of tyrosine phosphorylation and one capable of S/T phosphorylation (Walte et al., 2013). Therefore, upon binding to GSK3, SteE might mimic any of these effects, inducing a GSK3 conformational change that leads to a relaxed substrate specificity that enables tyrosine phosphorylation.

Another possibility is that mature GSK3 is intrinsically competent at phosphorylating tyrosines when such a residue (which is a more active nucleophile than a serine or threonine) is placed in the active site. However, *trans* tyrosine phosphorylation by GSK3 might not be detected in non-infected cells because of phosphatases and/or substrate specificity. Because GSK3 does not constitutively interact with STAT3, and the residues flanking Y705 on STAT3 do not conform to the canonical substrate recognition requirements of GSK3 (S/TXXX(p)S/T), it is highly likely that SteE functions as an adaptor to mediate STAT3 recognition and help position Y705 of STAT3 in the active site of GSK3. A direct interaction between SteE and STAT3 could explain why STAT3 is a substrate and why phosphorylated STAT3 remains detected in the GSK3-SteE complex rather than becomes immediately dissociated.

SteE is also a substrate of GSK3, and GFP-SteE^T91A/S141A/Y143F^ was unable to induce STAT3 activation. SteE contains a classical SH2 (Src homology 2)-binding motif (p)Y_143_XXQ, and experimentally we showed that Y143 was phosphorylated in a GSK3-dependent manner. Because STAT3 contains an SH2 domain, which mediates interactions with phosphorylated tyrosine residues, it is tempting to speculate that one function of GSK3-mediated SteE phosphorylation is to facilitate the interaction between SteE and STAT3. This is supported by our finding that SteE interacted with STAT3 only in the presence of GSK3. Furthermore, the region around the YXXQ motif of SteE resembles the cytoplasmic domain of the cytokine receptor common chain gp130 and interacts with STAT3 (Gibbs et al., 2019). Interestingly, SteE also contains a classical GSK3-phosphorylation motif, S_102_LPV(p)SP, and S106 is phosphorylated independently of GSK3. Even though we did not detect phosphorylation of S102 within this canonical GSK3 phosphorylation motif, the sequence could help mediate an interaction between GSK3 and SteE.

Together, our findings support a model in which GSK3 interacts with and phosphorylates SteE. This prevents the degradation of translocated SteE and promotes the formation of the SteE-GSK3-STAT3 complex. Within this complex, phosphorylated SteE then licenses GSK3 to phosphorylate the non-canonical substrate STAT3 on Y705 (Figure 6). Information on the conformational changes that GSK3 undergoes when bound by SteE awaits the molecular and structural determination of a SteE-GSK3-STAT3 complex. Whether the GSK3-SteE complex has other targets and whether this involves phosphorylation of tyrosine residues should now be explored.

The canonical substrates and functions of GSK3 are vast; more than 50 substrates have been reported, and GSK3 functions in regulating glucose homeostasis, cell proliferation, apoptosis, and cytokine signaling (Beurel et al., 2015, Cormier and Woodgett, 2017). Because only a fraction of GSK3 is bound by SteE, it is unlikely that its canonical functions involving S/T phosphorylation are perturbed. Whereas STAT3 is not described as a direct substrate, inhibition of GSK3β after stimulation with IFN-γ, IL-6, or IFN-α reduces the phosphorylation of STAT3 on Y705 (Beurel and Jope, 2008), and GSK3β modifies STAT3 phosphorylation in esophageal squamous cell carcinoma (Gao et al., 2017). During cytokine stimulation, GSK3β might promote an interaction between STAT3 and membrane tyrosine kinases. However, the possibility that a host chaperone-mediated switch in GSK3 substrate and amino acid specificity occurs, and whether this represents a broader mechanism in the regulation of this constitutively active kinase, deserves investigation.

Physiologically, *Salmonella*-induced STAT3 activation is reported to create a vacuole that is more permissive for *Salmonella* replication (Hannemann et al., 2013), and *steE* mutant bacteria have a severe replication defect *in vivo* (Lawley et al., 2006, Niemann et al., 2011). In addition, a new study in this issue reveals that SteE-mediated polarization of granuloma macrophages promotes persistence (Pham et al., 2019). The altered metabolic state of M2 macrophages might also support the persistence of intracellular pathogens, which in the case of *Salmonella* appears dependent on the host transcription factor PPARδ (Eisele et al., 2013, Xavier et al., 2013). Indeed, several other pathogens, including *Mycobacterium tuberculosis* (Huang et al., 2018, Sahu et al., 2017), *Coxiella* (Benoit et al., 2008), *Francisella* (Shirey et al., 2008), and *Brucella* (Kerrinnes et al., 2018), drive an anti-inflammatory M2-like host response. Whereas GRA18 from *Toxoplasma gondii* interacts with GSK3 and promotes transcription of anti-inflammatory genes, the mechanism is likely to be different because GRA18 functions in a β-catenin-dependent fashion (He et al., 2018). Overall, these findings suggest that there has been evolutionary pressure for intracellular pathogens to drive a more permissive and anti-inflammatory environment. In the case of *Salmonella* infection, macrophage polarization requires the concerted action of effectors that dampen pro-inflammatory signals and SteE-mediated anti-inflammatory activities (Stapels et al., 2018). This probably explains why SteE-mediated IL-10 production (Jaslow et al., 2018) is not sufficient to drive bystander cells into an M2-like state. Finally, the exploitation of GSK3 by SteE, as compared with driving STAT3 activation via canonical signaling, most likely renders SteE-mediated STAT3 activation recalcitrant to negative regulation by SOCS (suppressor of cytokine signaling) proteins, which inhibit JAK proteins (Croker et al., 2008).

In conclusion, our findings reveal a tightly regulated mechanism where a host S/T kinase is exploited to first phosphorylate and activate the virulence factor and then drive the host kinase to phosphorylate a non-canonical substrate on a tyrosine residue. Effector proteins have been previously described to carry out conventional eukaryotic post-translational modifications, to exhibit new enzymatic activities that result in irreversible and novel post-translational modifications (for example, arginine-GlcNAcylation), and to co-opt host enzymes. Our work described here provides another and more sophisticated effector mechanism: a protein that alters the amino acid and substrate specificity of a host kinase.

## STAR★Methods

### Key Resources Table

**Table:** 

REAGENT or RESOURCE	SOURCE	IDENTIFIER
**Antibodies**		

TruStain FcX™ (anti-mouse CD16/32)	BioLegend	Cat#101319; RRID: AB_1574973
Rat anti CD124 (IL-4Rα, clone IL4R-M1)	BD biosciences	Cat#551853; RRID: AB_394274
Goat anti CSA-1	BacTrace	Cat#01-91-99
Rabbit monoclonal anti pY705-STAT3	Cell Signaling	Cat#9145S; RRID: AB_2491009
Rat anti HA (Clone 3F10)	Roche	Cat#11867423001; RRID: AB_390918
Rabbit anti HA (HRP-conjugated)	R&D Systems	Cat#HAM0601
Rabbit anti HA	Sigma	Cat#H6908; RRID: AB_260070
Mouse anti HA.11	BioLegend	Cat#901503
Rabbit anti GFP	Life Technologies	Cat#G10362; RRID: AB_2536526
Mouse anti DnaK	Enzo	Cat#ADI-SPA-880-D; RRID: AB_2039064
Mouse anti Tubulin beta	DSHB	Cat#E7; RRID: AB_528499
Rabbit anti Actin	Sigma	Cat#A2066; RRID: AB_476693
Rabbit anti STAT3	Cell Signaling	Cat#12640S; RRID: AB_2629499
Rabbit anti STAT6	Cell Signaling	Cat#9362S; RRID: AB_2271211
Rabbit anti STAT6 pY641	Cell Signaling	Cat#56554S; RRID: AB_2799514
Rabbit anti His (HRP-conjugated)	Abcam	Cat#ab1187; RRID: AB_298652
Rabbit anti GSK3	Cell Signaling	Cat#5676S; RRID: AB_10547140
Rabbit anti FLAG	Sigma	Cat#F7425; RRID: AB_439687
Rabbit anti HSPA1L	Abcam	Cat#ab154409
Rabbit anti Thiophosphate ester	Abcam	Cat#ab92570; RRID: AB_10562142
Goat anti Rabbit	Agilent (Dako)	Cat#P0448; RRID: AB_2617138
Goat anti Mouse	Agilent (Dako)	Cat#P0447; RRID: AB_2617137

**Bacterial and Virus Strains**		

*Salmonella enterica* serovar Typhimurium, strain 14028s	Gift from David Holden (Jennings et al., 2018)	Proteome ID: UP000002695
*steE* mutant *Salmonella* (14028)	This study	N/A
*sseL* mutant *Salmonella* (14028)	Gift from David Holden (Mesquita et al., 2013)	N/A
*Salmonella enterica* serovar Typhimurium, strain SL1344	Gift from David Holden (Stapels et al., 2018)	N/A
*steE* mutant *Salmonella* (SL1344)	This study	N/A
WT or *steE* mutant *Salmonella* (14028) carrying pFCcGi	Stapels et al., 2018	Ν/Α
WT or *steE* mutant *Salmonella* carrying empty pWSK29 or pWSK29 for HA-tagged-SteE expression	This study	N/A
WT *Salmonella* carrying pWSK29 for HA-tagged-PipB expression	Gift from David Holden	N/A
*sseL* mutant *Salmonella* carrying pWSK29 for HA-tagged-SseL expression	Gift from David Holden (Mesquita et al., 2013)	N/A

**Chemicals, Peptides, and Recombinant Proteins**		

IL-4	Peprotech	Cat#214-14
IL-10	Peprotech	Cat#210-10
CHIR-99021	SelleckChem	Cat#S2924
Bosutinib	ApexBio	Cat#A2149
Cerdulatinib	ApexBio	Cat#B8023
Dasatinib	ApexBio	Cat#A3017
Erlotinib Hydrochloride	ApexBio	Cat#A8234
Imatinib	ApexBio	Cat#B2171
Midostaurin	ApexBio	Cat#B3709
PF-431396	ApexBio	Cat#A8692
PRT062607	ApexBio	Cat#A3736
Saracatinib	ApexBio	Cat#A2133
Tofacitinib	ApexBio	Cat#A4135
ATP	ThermoFisher	Cat#R0441
6-PhEt-ATP	BioLog, Life Science Institute	Cat#P012; CAS#181705-62-4
6-PhEt-ATPγS	BioLog, Life Science Institute	Cat#P026; CAS#944834-43-9
p-Nitrobenzyl mesylate (PNBM)	Abcam	Cat#ab138910
Lipofectamine 2000	Life Technologies	Cat#11668019
GenMute	Signagen	Cat#SL100568-PMG
Mouse serum	Sigma	Cat#S7273
GFP-TRAP	Pierce	Cat#gta-100
Pierce Anti-HA Agarose	ThermoFisher	Cat#26181
PhosSTOP™	Roche	Cat#4906837001
cOmplete™, Mini, EDTA-free Protease Inhibitor Cocktail	Roche	Cat#4693159001
HisPur™ Ni-NTA resin	ThermoFisher	Cat#88222
Recombinant Human His-GSK3β	This study	N/A
Recombinant His-MBP	This study	N/A
Recombinant His-MBP-SteEΔN20	This study	N/A
Recombinant Human His-STAT3	This study	N/A
Recombinant Human GST-STAT3	Abcam	Cat#ab43618

**Critical Commercial Assays**		

Pro-Q® Diamond Phosphoprotein Gel Stain	Invitrogen	Cat#P33300
Dual-luciferase reporter assay system	Promega	Cat#E1980
Cignal STAT3 Reporter (luc) Kit	QIAGEN	Cat#CCS-9028L
Mouse macrophage nucleofector kit	Lonza	Cat#VPA-1009
LIVE/DEAD Fixable Blue stain	Invitrogen	Cat#L34961

**Experimental Models: Cell Lines**		

293ET	Gift from Felix Randow	RRID: CVCL_6996
HeLa	American Tissue Culture Collection	RRID: CVCL_0030
GSK3α^−/−^ 293ET cells	This study	N/A
GSK3β^−/−^ 293ET cells	This study	N/A
GSK3αβ^−/−^ 293ET cells	This study	N/A

**Experimental Models: Organisms/Strains**		

C57BL/6 *Nramp*^+/+^ mice	In house colony	Jacobson, 2002
C57BL/6 mice for bone marrow derived macrophages	Charles River	N/A
*E. coli* BL21	Cherepanov, 2007	N/A
Sf9 insect cells	ThermoFisher	Cat#11496015

**Oligonucleotides**		

Primer sequences	See Table S1	N/A
guideRNA to target GSK3α GCTGCCGCCGGGTCCACCCC	This study	N/A
guideRNA to target GSK3β GTCCTGCAATACTTTCTTGA	This study	N/A
ON-TARGETplus STAT3 siRNA	Dharmacon	Cat#L-040794-01-0005
Non-targeting control siRNA	Dharmacon	Cat#D-001810-01-05

**Recombinant DNA**		

pX330	Cong et al., 2013	RRID: Addgene_42230
M5p and related plasmids	Gift from Felix Randow (Randow and Sale, 2006)	N/A
pTCMV	Jennings et al., 2017	N/A
pET49	Martino et al., 2018	N/A
pACEBac	Gift from Katrin Rittinger	N/A

**Software and Algorithms**		

Prism	GraphPad Version 8	https://www.graphpad.com/scientific-software/prism/
Excel	Microsoft	Version 16.16.5
Image Lab	BioRad	https://www.bio-rad.com/en-uk/product/image-lab-software?ID=KRE6P5E8Z
FlowJo	TreeStar	https://www.flowjo.com/

### Lead Contact and Materials Availability

Further information and requests for resources and reagents should be directed to and will be fulfilled by the Lead Contact, Teresa L.M. Thurston (t.thurston@imperial.ac.uk).

### Experimental Models and Subject Details

#### Cell Culture

HEK293ET cells and HeLa cells (Gifts from Felix Randow, MRC-LMB) were maintained in Dulbecco’s modified eagle medium (DMEM; Sigma) supplemented with 10% heat-inactivated fetal calf serum (FCS; GIBCO, Life Technologies) at 37°C in 5% CO_2_.

Primary bone-marrow-derived macrophages (pBMDMs) were prepared from the tibia and femur of 6 to 8-week old female C57BL/6 mice (Charles River), in accordance with a UK Home Office Project License in a Home Office designated facility. Red blood cells were lysed in 0.83% NH_4_Cl for 3 min, and then the remaining progenitor cells were cultured in DMEM containing 20% L929 culture supernatant (LCM), 10% FCS, 10 mM HEPES (Sigma), 1 mM sodium pyruvate (Sigma), 0.05 mM beta-mercaptoethanol (Sigma), 100 U/mL penicillin (Sigma) and 100 μg/mL streptomycin (Sigma) at 37°C in 5% CO_2_. After three days, fresh medium was added and the differentiated pBMDMs were harvested at day seven and seeded without LCM or antibiotics in tissue culture treated 6-well or 10 cm dishes.

#### Animal Strains and Infection Conditions

C57BL/6 *Nramp*^+/+^ mice were derived as described previously and obtained from an in-house colony (Jacobson, 2002). Female and male mice 6-12 weeks old were infected with either 10^3^ CFU WT SL1344 or Δ*steE* SL1344 in 200 μL sterile PBS via intraperitoneal inoculation and analyzed at 10 days post-infection. Organs were collected, weighted, and either homogenized in PBS for CFU enumeration or used to make single cell suspensions for flow cytometric analysis.

#### Mouse Ethics Statement

Animal experiments were performed in accordance with NIH guidelines, the Animal Welfare Act, and US federal law and approved by the Stanford University Administrative Panel on Laboratory Animal Care (APLAC) and overseen by the Institutional Animal Care and Use Committee (IACUC) under Protocol ID 12826. Prior to experimentation, mice were given at least one week to acclimatise to the Stanford Animal Biohazard Research Facility.

### Methods Details

#### Analysis of Splenic Mononuclear Phagocytes

Splenic single-cell suspensions were incubated in Fc Block (TruStain fcX anti-mouse CD16/32, Biolegend) for 15 min on ice and washed with PBS. Cells were stained on ice for 25 min in PBS with a cocktail of Live/Dead Fixable Blue Viability Dye (Invitrogen) and antibodies for surface antigens. Cells were washed with FACS buffer (PBS containing 2% FBS and 2 mM EDTA), followed by fixation for 15 min with Cytofix/Cytoperm solution (BD Biosciences). Cells were washed twice with Perm/Wash buffer (BD Biosciences) and stained for intracellular *Salmonella* using CSA-1 antibody (KPL). After washing, cells were resuspended in FACS buffer and analyzed on a LSRFortessa cytometer (Becton Dickinson). Data were acquired with DIVA software (BD Biosciences) and analyzed using FlowJo software (TreeStar). Splenocytes were gated for live, singlet, CD19^-^CD3^-^NK1.1^-^ cells and then further gated to select CD11b^+^MHCII^+^F4/80^+^, Ly6G-negative mononuclear phagocytes. Intracellular staining with anti-*Salmonella* FITC antibody was used to identify infected from non-infected cells. IL-4Rα levels were measured as the median fluorescent intensity.

#### DNA Plasmids

M5P or closely related plasmids (gift from Dr Randow, MRC-LMB) were used for both transient transfection and for transduction with recombinant MLV to produce stable expression of proteins in mammalian cells (Randow and Sale, 2006). Alternatively, 200-400 ng of pTCMV-GFP-[gene] plasmids were used per 24 well for transient expression in 293ET cells. Open reading frames encoding human GSK3α (UniProt: P49840) and GSK3β (UniProt: P49841 were amplified by PCR from 293ET cDNA. During the course of our studies we noted that the start methionine for SteE is differentially annotated on uniprot depending on the *Salmonella* strain. Based on *in silico* analysis of the Shine-Dalgarno sequence, predicted translation initiation rates and experimental analysis of the protein by immunoblot, this study uses a sequence corresponding to amino acids 25 to 181 from the annotated version of SteE (STM2585, UniProt: Q8ZN17). This corresponds to the start methionine in the annotated versions of SteE with UniProt: A0A455RQ54, A0A484YIU7, A0A0U1H469 and A0A447N637. This sequence was amplified from *Salmonella* genomic DNA (strain 14028s) and represents the wild-type form of SteE used in this paper (Figure 4C). Any truncations or point mutations were introduced by PCR-mediated mutagenesis. pET49-His-MBP-SteEΔN20 (vector source (Martino et al., 2018)) was used for protein expression in BL21 *E. coli* and pACEBac-His-GSK3β and pACEBac-His-STAT3 expression vectors (vectors were a gift from Dr Katrin Rittinger) were used for the generation of recombinant human GSK3β and STAT3 (UniProt: P40763) from SF9 insect cells.

#### DNA and RNA Transfections

Plasmid DNA was transfected using Lipofectamine 2000 (Life Technologies) as per the manufacturer’s instructions. ON-TARGETplus STAT3 siRNA (Dharmacon, L-040794-01-0005) or ON-TARGETplus non-targeting control siRNA (Dharmacon, D-001810-01-05) was transfected using either GenMute (Signagen) or the mouse macrophage nucleofector kit (Lonza, VPA-1009) according to the manufacturer’s instructions using a final siRNA concentration of 50 nM. Cells were infected two days after siRNA treatment.

#### Flow Cytometry

Macrophages, infected for 17 h as described above, were fixed for 15 min in 3% PFA/PBS and immunolabelled for extracellular IL-4Rα (BD biosciences, clone mIL4R-M1) in 10% horse serum / PBS for 30 min before analysis on a BD Fortessa flow cytometer. Gates were set to identify the infected macrophages that contained intracellular bacteria carrying the plasmid pFCcGi, which encodes constitutively expressed mCherry and arabinose-inducible GFP, from the non-infected bystanders. The percentage of IL-4Rα macrophages was then determined in each population using FlowJo software.

#### Fluorescence-Activated Cell Sorting

pBMDMs, infected for 18 h as described above, were washed twice in PBS before being detached from the plates by scraping in cold PBS. Live cells were then stained for extracellular IL-4Rα using a BV405-conjugated antibody (BD biosciences clone mIL4R-M1) in 10% horse serum / PBS for 30 min before FACS was used to sort cells under continuous cooling to 4°C on a BD FACS Aria III into three populations: non-infected bystanders, cells containing mCherry and GFP-positive bacteria that were IL-4Rα negative and cells containing mCherry and GFP-positive bacteria that were IL-4Rα positive. The gating strategy was set to exclude apoptotic macrophages and doublets and only include infected macrophages that had a similar bacterial burden (based on mCherry intensity). The collected cells were then lysed in SDS lysis buffer and analyzed by immunoblotting.

#### Generation of CrispR/Cas9 Knockout Cell Lines

293ET cells were transiently transfected with pX330 (Cong et al., 2013) containing a guideRNA to target GSK3α (GCTGCCGCCGGGTCCACCCC) or GSK3β (GTCCTGCAATACTTTCTTGA) or both plasmids to create the double knockout cell line. After 24 h, cells were seeded into 96 well plates at 0.3 cells / well. Single clones were screened by immunoblotting with anti-GSK3 antibody. In addition, the target gene was sequenced to confirm gene-editing.

#### Immunoprecipitation

The indicated cells were lysed in lysis buffer (10% glycerol, 20 mM Tris Cl pH 7.4, 150 mM NaCl, 0.1% Triton X-100) supplemented with protease inhibitors (1 mM PMSF, 1 mM benzamidine, 1 μg/mL aprotinin, 5 μg/mL leupeptin) and phosSTOP (Roche) and clarified by centrifugation at 17,000 x *g* for 10 min at 4°C. GFP-TRAP (ChromoTek) or anti-HA beads (ThermoFisher) were equilibrated in cold lysis buffer and incubated with the lysate for at least 2 h at 4°C with rotation. Beads were then washed three times with 1 mL lysis buffer and bound proteins were eluted by the addition of SDS loading buffer. The experiments used to analyze the interaction partners of SteE by mass spectrometry (LC-MS-MS) contained 1 mM DTT in the buffer and phosSTOP was excluded. For the identification of proteins and phosphorylated amino acids by mass spectrometry (LC-MS-MS), beads with bound protein from triplicate experiments were sent for analysis at the Institute of Biochemistry and Biophysics (IBB) at the Polish Academy of Sciences, Warsaw, Poland. Acquired spectra were compared to a protein sequence database (*S*. Typhimurium 14028s, Uniprot; *Homo sapiens*, Swiss-Prot) using the MASCOT search engine.

#### Kinase Assays

293ET stably expressing GFP, GFP-GSK3α and GFP-GSK3β were seeded in T175 flasks and grown to confluency. Alternatively, 293ET cells seeded in a 6-well format, were transiently transfected with 1 μg ptCMV.GFP or 1 μg ptCMV.GFP-SteE for 24 h. GFP-tagged proteins were immunoprecipitated on beads as described above, but without performing the elution step. After two washes in kinase buffer (25 mM HEPES pH 7.4, 25 mM MgCl2, 1 mM tris[2-carboxyethyl]phosphine [TCEP], 25 mM β-glycerophosphate, 0.1 nM NaVO_3_, 0.5 mM NaF, 100 nM Okadaic Acid), the beads were resuspended in 50 μL kinase buffer containing 1 mM ATP (ThermoFisher), 1.6 μg His-MBP (this study) or 1.6 μg His-MBP-SteEΔN20 (this study), 0.4 μg GST-STAT3 (Abcam) or 0.4 μg His-STAT3 (this study), as indicated. The reactions were carried out at 30°C with agitation at 600 RPM in a PCMT Grant-bio thermomixer for 30 min and stopped by adding 5x SDS loading buffer. Thereafter, the samples were boiled at 95°C for 5 min, centrifuged at 1500 x *g* for 1 min and the eluted proteins were subjected to immunoblot analysis.

Alternatively, kinase assays were performed in Kinase Buffer with the following recombinant proteins, 5 μg His-GSK3β (this study), 12.5 μg His-MBP or His-MBP-SteEΔN20 (this study), together with 0.4 μg GST-STAT3 (Abcam), with or without 1 mM ATP as indicated. After 30 min the reaction was terminated with the addition of 5x SDS loading buffer. The samples were analyzed by SDS-PAGE and immunoblotting as well as with Pro-Q® Diamond Phosphoprotein Gel Stain as per the manufactures’ recommended protocol (Invitrogen). After analysis on a ChemiDoc Imaging System (BioRad) the gel was then stained with Coomassie.

For experiments involving the use of a bulky ATP analog, site directed mutagenesis was used to expand the ATP binding pocket of GSK3α, by substituting Leucine 195 to Glycine. 293ET cells were seeded in 6-well plates and transfected with 800 ng ptCMV.GFP, 1.2 μg m6pBLAST:GFP-GSK3α or 1.2 μg m6pBLAST:GFP-GSK3α^L195G^ for 48 h. The kinase assay was performed as described above but using 0.4 mΜ ATP or 0.4 mM 6-PhEt-ATP or 0.4 mM 6-PhEt-ATPγS (BioLog, Life Science Institute). For experiments involving the 6-PhEt-ATPγS analog, the kinase reaction was performed using an equimolar amount of GST-STAT3 and His-MBP-SteEΔN20 and the kinase reaction was allowed to proceed as described above but for 1 h. Where indicated, alkylation was performed by incubating the kinase assay samples in 2.5 mM p-Nitrobenzyl mesylate (PNBM; Abcam) for 1 h at room temperature. Thereafter, the reaction was stopped by adding 5x SDS loading buffer, samples were boiled at 95°C for 5 min and subjected to immunoblot analysis.

#### Microscopy

HeLa cells, grown on glass coverslips, were infected as above and following two PBS washes were fixed in 4% paraformaldehyde in PBS for 20 min. Cells were then permeabilized and blocked in either 0.1% Triton X-100 / PBS / 10% horse serum or 0.1% Saponin / PBS / 10% horse serum before incubating the coverslips with primary and then secondary antibodies (Alexa-Flour, Invitrogen) with the addition of 0.5 μg/mL diamidino-2-phenylindole (DAPI, Invitrogen) for 1 h at room temperature. Samples were then mounted onto glass slides using Aqua-Poly/Mount (Polysciences, Inc.) and visualized on an LSM 710 inverted confocal microscope (Zeiss GmbH) with a x 63, 1.4 numerical aperture objective.

#### Protein Purification

For the bacterial expression of recombinant SteE, pET49-His-MBP-SteEΔN20 was transformed into BL21 PC2 *E. coli* competent cells (Cherepanov, 2007). The cells were grown in LB broth at 37°C to an OD_600_ of 0.6 to 0.8, after which protein expression was induced with 1 mM isopropyl β-D-1-thiogalactopyranoside (IPTG) overnight at 18°C. The cells were harvested by centrifugation and cell pellets were suspended in lysis buffer 1 (50 mM Tris-Cl pH 8, 500 mM NaCl, 0.5 mM TCEP, 10% [v/v] glycerol and protease inhibitors (Roche)) and lysed by sonication using a Bandelin Sonoplus sonicator (50% amplitude, 30 s pulse on, 10 s pulse off) for 5 min. The cell lysate was clarified at 38000 x *g* for 30 min at 4°C to recover the protein-containing supernatant, which was then purified using HisPur Ni-NTA resin (ThermoFisher).

Recombinant GSK3β and STAT3 were expressed in SF9 insect cells (ThermoFisher Scientific 11496015), according to the previously described protocol (Martino et al., 2016). For protein purification, cell pellets were resuspended in lysis buffer (50 mM Tris-Cl pH 8, 300 mM NaCl, 20 mM Imidazole, 10% [v/v] glycerol, 0.5 mM TCEP, 1 mM PMSF, 5 μg/μl DNaseI, 10 mM MgCl_2_ and protease cocktail inhibitor tablets [Roche]) and lysed with 0.5% Triton X-100. The cell lysate was clarified by centrifugation in order to recover the protein-containing supernatant, which was then purified using HisPur Ni-NTA resin.

The HisPur Ni-NTA resin was equilibrated with binding buffer (50 mM Tris-CI pH 7.4, 500 mM NaCl, 20 mM Imidazole, 0.5 mM TCEP) before the supernatant was passed through the resin and washed thoroughly with binding buffer to remove contaminants and weak binding proteins. The protein was then eluted with 50 mM Tris-CI pH 7.4, 500 mM NaCl, 500 mM Imidazole, 0.5 mM TCEP and dialysed overnight in 20 mM Tris-CI pH 7.4, 200 mM NaCl and 0.5 mM TCEP. Protein samples were collected, ran on an SDS-PAGE gel and flash-frozen with liquid nitrogen to be stored at −80°C until use.

#### *Salmonella* Infection

*Salmonella enterica* serovar Typhimurium, strain 14028s, and its isogenic mutants, were used for all cell culture studies. *steE* mutant *Salmonella* was made via lambda-red recombination (Datsenko and Wanner, 2000) and the wild-type strain, *sseL* mutant *Salmonella* (Mesquita et al., 2013) and other strains were a kind gift from Prof. David Holden (Imperial College London). Strains carrying pWSK29 for HA-tagged effector expression or pFCcGi (Figueira et al., 2013) for mCherry and GFP fluorescence, were grown with 50 μg/mL carbenicillin. 50 μg/mL kanamycin was added for the culture of bacterial mutant strains.

For SPI-1 induced infection of HeLa or 293ET cells, bacteria were grown overnight in Luria broth (LB) and sub-cultured (1:33) in fresh LB for 3.5 h prior to infection at 37°C. Cells seeded in 24-well or 6-well format were infected with 10 μL or 23 μL of *Salmonella*, respectively for 10 min at 37°C. Cells seeded in 10 cm dishes or T175 flasks were infected with 300 μL or 700 μL of bacteria. After two PBS washes, cells were incubated in 100 μg/mL gentamycin for 1-2 h and 20 μg/mL gentamycin thereafter.

For the infection of primary macrophages, bacteria were grown in minimal MgMES medium (170 mM 2-(N-morpholino)ethanesulfonic acid (MES) at pH 5, 5 mM KCl, 7.5 mM (NH_4_)_2_SO_4_, 0.5 mM K_2_SO_4_, 1 mM KH_2_PO_4_, 8 mM MgCl_2_, 38 mM glycerol and 0.1% casamino acids). 0.2% w/vol L-arabinose was included for the induction of GFP in strains carrying plasmid pFCcGi. Bacteria that had been opsonised with 8% mouse serum (Sigma) for 20 min were added to macrophages at MOI of 5. Following centrifugation at 100 x *g* for 5 min the cells and bacteria were incubated for 25 min at 37°C with 5% CO_2_. Following two PBS washes to remove non-phagocytosed bacteria, cells were cultured with 50 μg/mL gentamycin for 1 h and 15 μg/mL thereafter.

#### *Salmonella In Vitro* Expression Assay

To test for protein expression of the HA-tagged SteE variants, *steE* mutant *Salmonella* carrying pWSK29 with either the long or short variant of SteE expressed under the ssaG promoter, were sub-cultured for 4 h in minimal MgMES pH 5 media and analyzed by immunoblot.

#### SDS-PAGE and Immunoblotting

To prepare whole cell lysates, cells were washed once in PBS and lysed in SDS loading buffer before sonication. For post nuclear supernatant (PNS) and pellet analysis, cells were washed once in PBS and lysed in Cell Lysis Buffer (10% glycerol, 20 mM Tris-Cl pH7.4, 150 mM NaCl, 0.1% Triton X-100) on ice for 10 min before clarification by centrifugation. SDS loading buffer was added to the PNS and pellet samples so that both fractions were in the same final volume. All samples were then heated to 95°C and separated by SDS-PAGE using either 8, 10 or 12% polyacrylamide denaturing gels, transferred to PVDF membrane (Millipore) and visualized by immunoblotting using ECL detection reagents (Dako) on a Chemidoc™ Touch Imaging System (Bio-Rad).

#### STAT3 Luciferase Reporter Assay

293ET cells were seeded into 24 well plates prior to transfection with 50 ng STAT3-responsive luciferase reporter plasmid kit (QIAGEN Cignal) and 250 ng of the indicated pTCMV-GFP-SteE construct. 24 h after transfection, luciferase activity in cell lysates was measured using the Dual Luciferase reporter assay system (Promega) on a Tecan Infinite 200 PRO plate reader. STAT3-dependent *Firefly* luciferase values were normalized to *Renilla* luciferase values and the fold change relative to GFP-expressing control cells was calculated.

### Quantification and Statistical Analysis

Data were tested for statistical significance with GraphPad Prism software. The number of replicates for each experiment and the statistical test performed are indicated in the figure legends. When analyzed, immunoblot band intensity was quantified using ImageLab software.

### Data and Code Availability

This study did not generate datasets or code.

## Supplemental Information

Document S1. Figures S1–S6 and Table S1

Document S2. Article plus Supplemental Information
