# Supplementary material for: Salmonella Effector SteE Converts the Mammalian Serine/Threonine Kinase GSK3 into a Tyrosine Kinase to Direct Macrophage Polarization
Source: Cell Host Microbe. 2020 Jan 8;27(1):41–53.e6. doi: 10.1016/j.chom.2019.11.002 (PMC6953433; doi:10.1016/j.chom.2019.11.002)
Supplement: Document S1. Figures S1–S6 and Table S1 [file mmc1.pdf]

Cell Host & Microbe, Volume *27*

Supplemental Information

Salmonella Effector SteE Converts the Mammalian Serine/Threonine Kinase GSK3 into a Tyrosine Kinase to Direct Macrophage Polarization

Ioanna Panagi, Elliott Jennings, Jingkun Zeng, Regina A. Günster, Cullum D. Stones, Hazel Mak, Enkai Jin, Daphne A.C. Stapels, Nur. Z. Subari, Trung H.M. Pham, Susan M. Brewer, Samantha Y.Q. Ong, Denise M. Monack, Sophie Helaine, and Teresa L.M. Thurston

Figure S1, related to Figure 1: FACS gating strategies

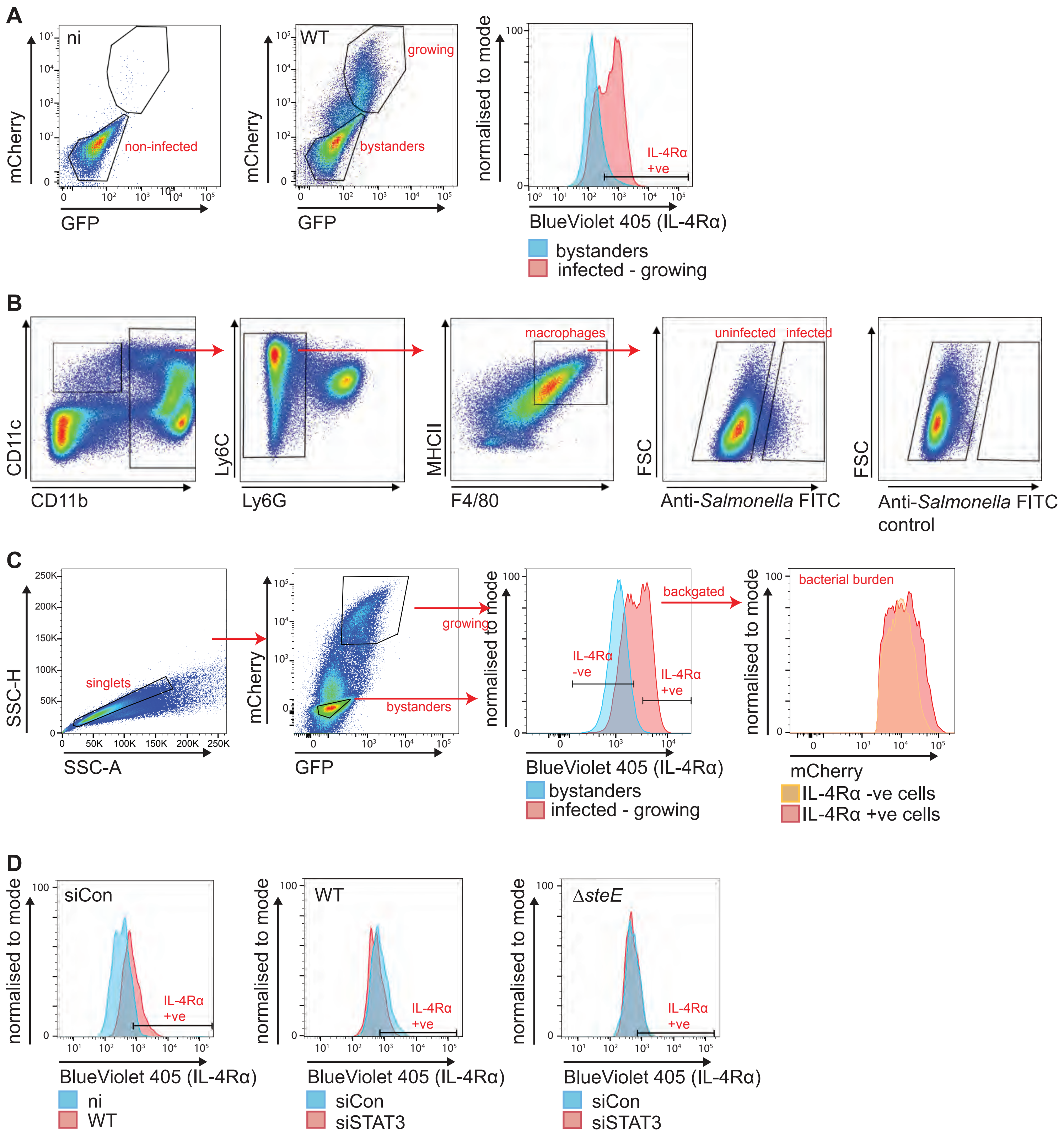

Figure S1, related to Figure 1: FACS gating strategies

A) Gating strategy used in Figure 1A for pBMDMs infected for 17 hours with WT or *steE* mutant *Salmonella* carrying the fluorescent plasmid pFCcGi. Non-infected (panel 1), non-infected bystanders and cells infected with growing bacteria (panel 2) were gated as shown. Panel 3 represents the gating strategy to select the percentage of IL-4Rα-positive cells.

B) The FACS plots show the gating strategy used to identify $CD11b^{+}MHCII^{+}F4/80^{+}$, Ly6G-negative mononuclear phagocytes that were non-infected or infected with *Samlonella*. The data are related to Figure 1B.

C) Gating strategy used to sort singlet pBMDMs (panel 1) into bystanders or infected cells that contained growing (high mCherry signal) WT *Salmonella* carrying pFCcGi (panel 2). Panel 3 shows the strategy to selects cells that were IL-4Rα-negative or -positive. Panel 4 shows a histogram representing the mCherry signal in the sorted IL-4Rα-negative or -positive populations from WT-infected pBMDMs in the infected – growing gate. The sorted bystander cells and cells with growing bacteria that were IL-4Rα negative or positive were then used for immunoblot analysis in Figure 1D.

D) Gating strategy used to identify the percentage of IL-4Rα-positive pBMDMs following treatment with control siRNA in non-infected and WT-infected cells (panel 1) as well WT (panel 2) or *steE* mutant *Salmonella* (panel 3) carrying pFCcGi in control or siSTAT3-treated pBMDMs from Figure 1E.

Figure S2, related to Figure 2: SteE interacts with the kinase domain of GSK3 and pY705-STAT3

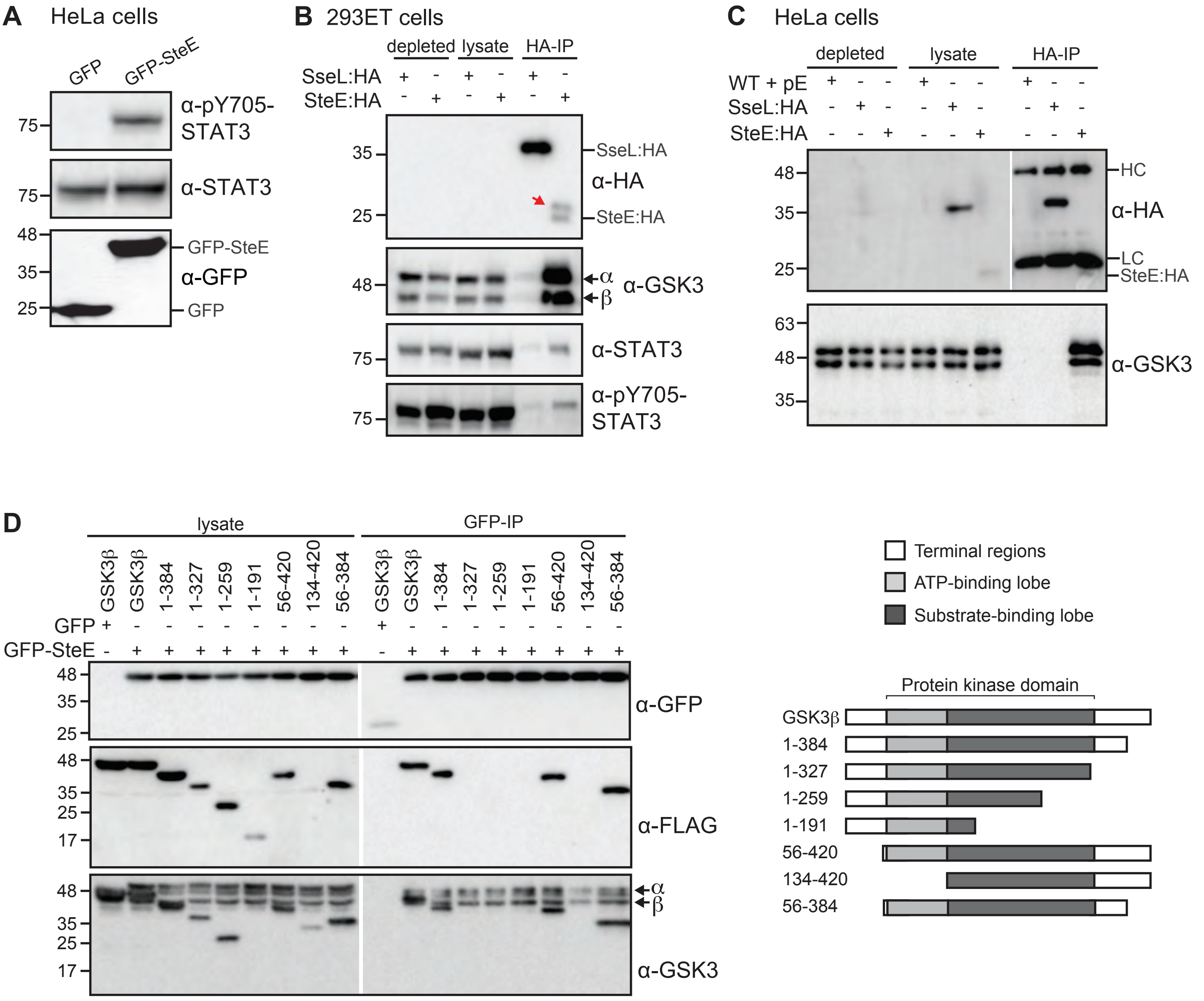

Figure S2, related to Figure 2: SteE interacts with the kinase domain of GSK3 and pY705-STAT3

(A) Whole cell lysates from HeLa cells transiently expressing GFP or GFP-SteE were analysed by immunoblot with antibodies against STAT3, pY705-STAT3 and GFP. Data are representative of three independent experiments.

(B) 293ET cells were infected with *steE* mutant *Salmonella* carrying pWSK29-SteE:HA or *sseL* mutant *Salmonella* carrying pWSK29-SseL:HA for 17 hours after which HA-tagged effectors were immunoprecipitated from cell lysates and assessed for their ability to bind endogenous GSK3, STAT3 or pY705-STAT3 as indicated. Immunoblots are representative of three independent experiments. The red arrow indicates a higher molecular weight form of SteE.

(C) HeLa cells infected with WT *Salmonella* carrying an empty plasmid (pE), *steE* mutant *Salmonella* carrying the pWSK29-SteE:HA plasmid or *sseL* mutant *Salmonella* carrying pWSK29-SseL:HA were lysed at 17 hours post infection and HA-tagged proteins immunoprecipitated using anti-HA agarose. Depleted lysates, lysates and HA-immunoprecipitated samples were then probed by immunoblot for HA and endogenous GSK3. Data represents three independent experiments. HC – heavy chain; LC – light chain.

(D) GFP or GFP-SteE was co-expressed in 293ET cells with the indicated FLAG-tagged GSK3 variants, followed by cell lysis and GFP immunoprecipitation. Lysate and IP samples were analysed by immunoblotting for GFP, FLAG or endogenous GSK3. A schematic of the GSK3 variants is shown. Data shown are representative of three repeats.

Figure S3, related to Figure 3: GSK3 kinase activity is required for *Salmonella* but not IL-10-induced STAT3 activation or macrophage M2 polarization

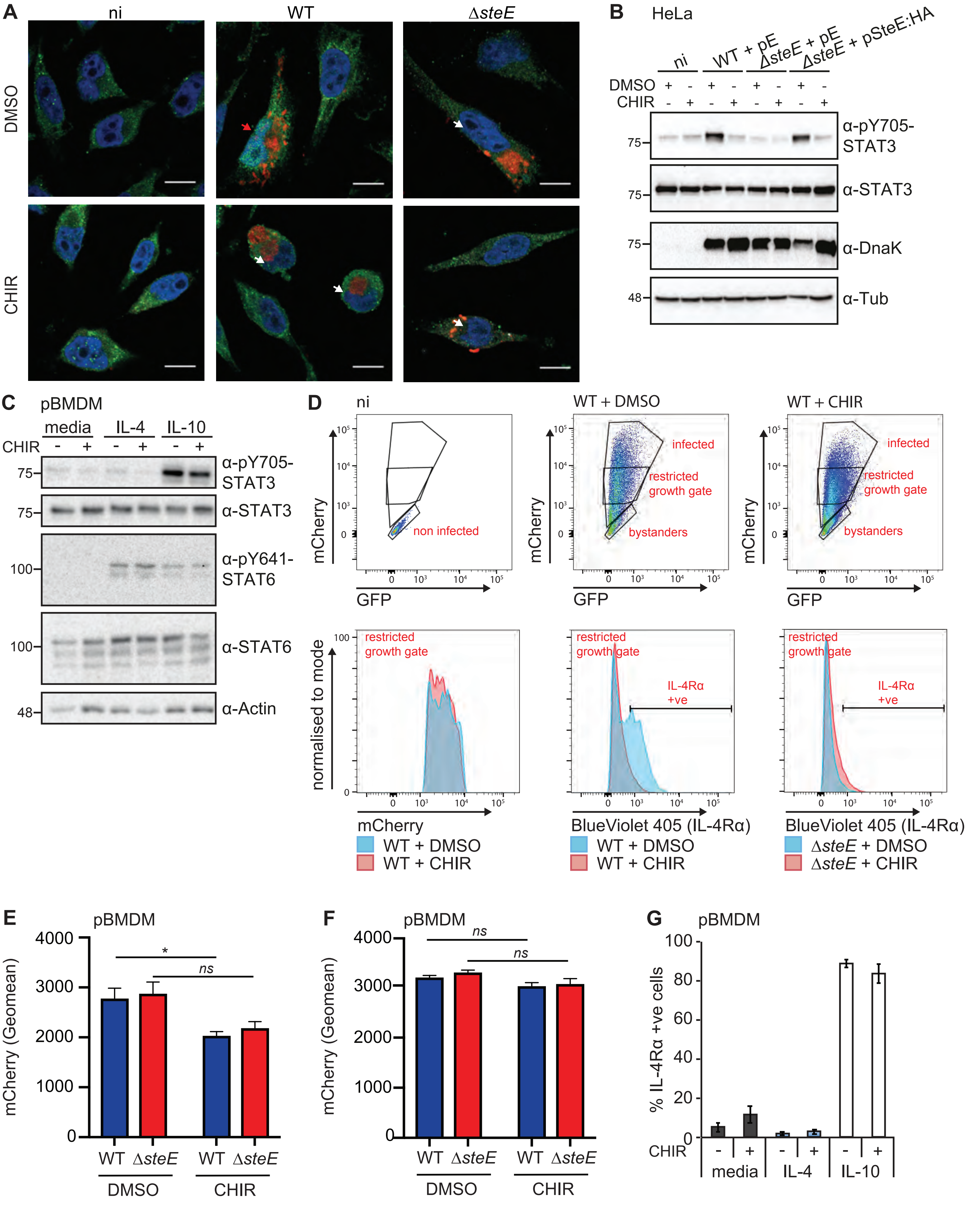

Figure S3, related to Figure 3: GSK3 kinase activity is required for *Salmonella* but not IL-10-induced STAT3 activation or macrophage M2 polarization

(A) HeLa cells were infected with the indicated *Salmonella* strains and treated with DMSO or 5 μM CHIR99021 from 1 hour post uptake. At 17 hours post infection, the cells were fixed, permeabilised then labelled for Y705-phosphorylated STAT3 (green), CSA1 (*Salmonella,* red) and DAPI (nucleus, blue). The red arrow indicates a cell where enriched nuclear pY705-STAT3 was detected, white arrows indicate infected cells showing background / diffuse cytosolic pY705-STAT3 signal, as quantified in Figure 3A. Scale bar, 10 μm.

(B) HeLa cells were infected with the indicated *Salmonella* strains for 17 hours and treated with DMSO or 5 μM CHIR99021 from 1 hour post uptake. Cell lysates were analysed by immunoblotting with antibodies against active STAT3 (pY705), STAT3, DnaK for a *Salmonella* infection control or tubulin (Tub) as a loading control. Immunoblots are representative of three independent experiments.

(C) pBMDMs were treated with either 20 mg/ml IL-4 or 20 mg/ml IL-10 for 17 hours in the presence or absence of 5 μM CHIR99021. Cell lysates were analysed by immunoblotting with antibodies against active STAT3 (pY705), STAT3, active STAT6 (pY641), STAT6 or actin as a loading control. Data are representative of two independent experiments.

(D) Gating strategy used in Figure 3C to identify non-infected, bystander and infected pBMDMs that had been infected for 17 hours with WT or *steE* mutant *Salmonella* carrying the fluorescent plasmid pFCcGi. A restricted growth gate was used for analysis of cells with a similar bacterial burden between DMSO and CHIR9902-treated samples. Representative histograms showing the mCherry fluorescence for WT-infected cells backgated on the restricted growth gate and IL-4Rα gating strategy for WT and *steE* mutant infected DMSO and CHIR9902-treated are shown in the lower panels.

(E) Analysis of mCherry geometric mean fluorescence in pBMDMs infected for 17 hours using the "infected" gate shown in (D). Mean and SEM of three independent experiments. *ns* – not significant, * $P < 0.05$, two-way ANOVA with Bonferroni's multiple comparisons tests.

(F) Analysis of mCherry geometric mean fluorescence in pBMDMs infected for 17 hours using the "restricted growth gate" as shown in (D). Mean and SEM of three independent experiments. *ns* – not significant, two-way ANOVA with Bonferroni's multiple comparisons tests.

(G) pBMDMs, treated as in (C) were analysed by flow cytometry for the percentage of IL-4Rα-positive cells. Mean and SEM of four independent experiments.

Figure S4, related for Figure 3: SteE:HA but not GFP-SteE is degraded in the absence of active GSK3

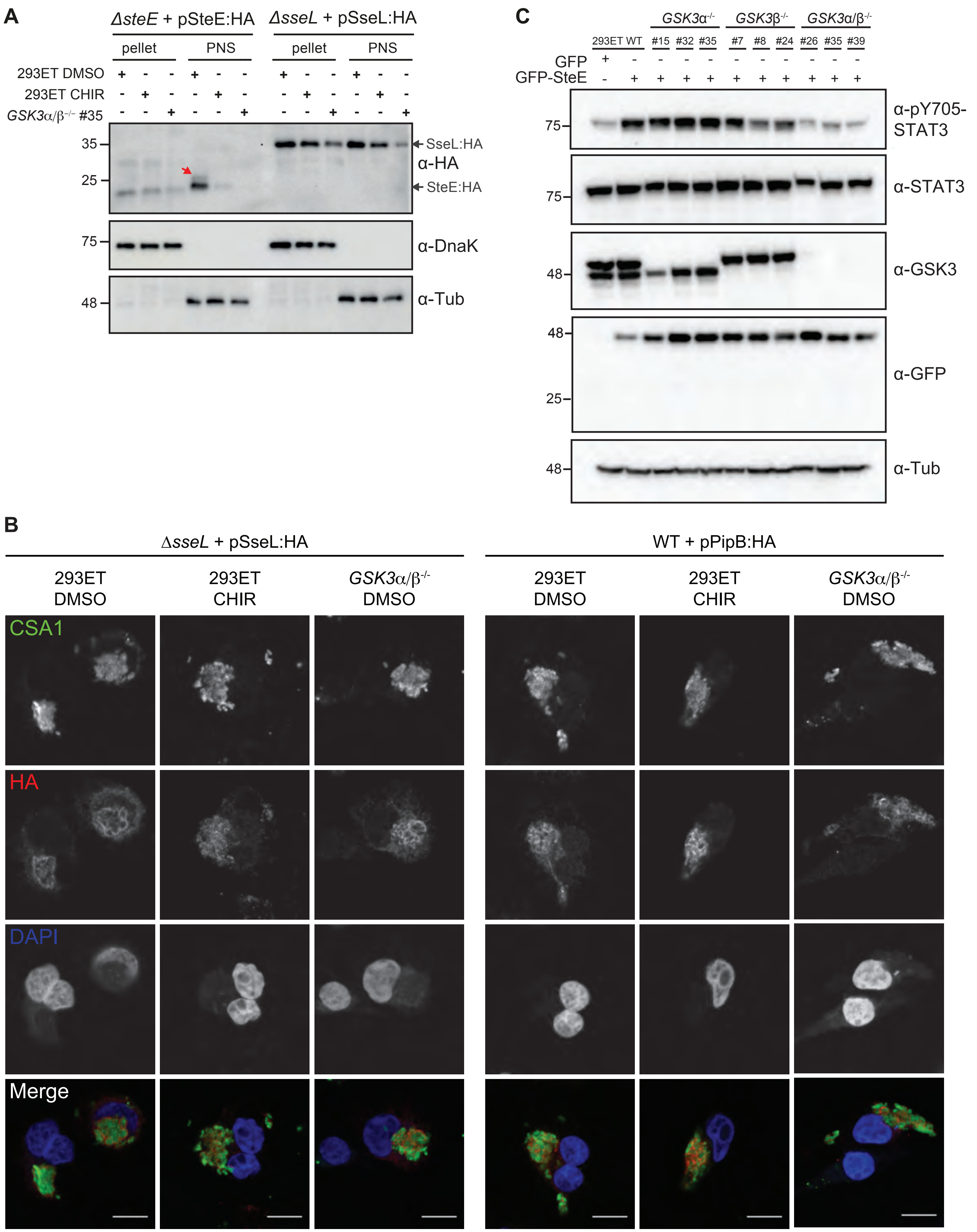

Figure S4, related to Figure 3: SteE:HA but not GFP-SteE is degraded in the absence of active GSK3

(A) Translocation of HA-tagged SteE or SseL T3SS effectors detected in cell pellet or post nuclear supernatant (PNS) after lysis of the indicated cell lines at 17 hours post infection. Where specified, cells were treated with 5 μM GSK3 inhibitor CHIR99021 or DMSO as vehicle control from 1 hour post infection. The red arrow indicates a higher molecular weight form of SteE. Data are representative of two repeats.

(B) Representative confocal micrographs of 293ET cells infected with the indicated *Salmonella* strains and treated with either DMSO or 5 μM GSK3 inhibitor CHIR99021 at 1 hour post infection. Samples were fixed at 17 hours post infection, permeabilised with saponin and labelled for CSA1 (*Salmonella*, green), HA (effector, red) and DAPI (nucleus, blue). Scale bar, 10 μm.

(C) GFP or GFP-SteE was transiently expressed in either WT 293ET cells or the indicated GSK3 knockout cell line clones and cell lysates were immunoblotted with the indicated antibodies. Immunoblots are representative of two experiments.

Figure S5, related for Figure 4: STAT3 and SteE are phosphorylated by a GSK3-containing complex

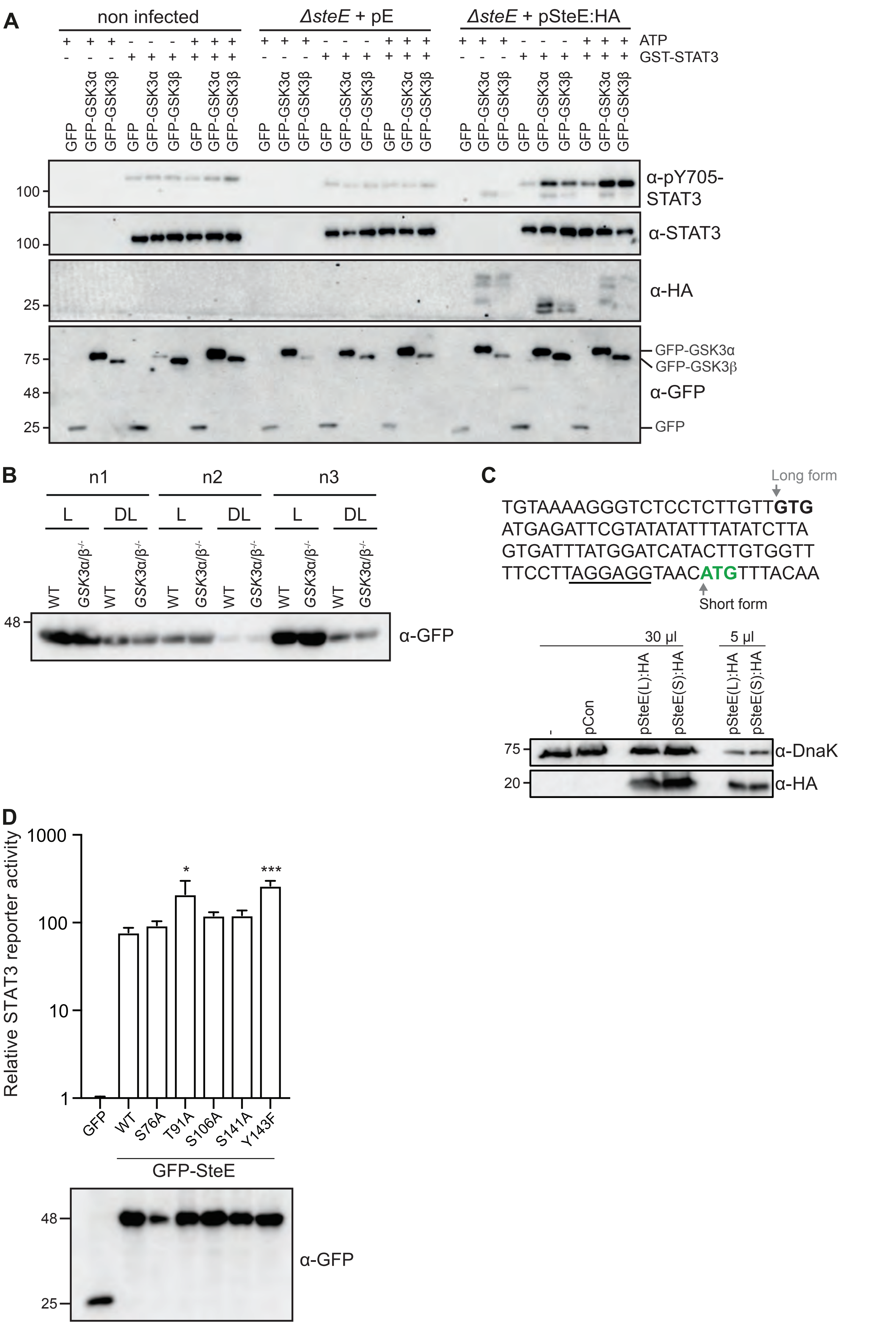

Figure S5, related to Figure 4: STAT3 and SteE are phosphorylated by a GSK3-containing complex

(A) 293ET cells stably expressing GFP, GFP-GSK3α or GFP-GSK3β were infected with the indicated *Salmonella* strains for 17 hours. Subsequently, the cells were lysed and GFP-tagged proteins were immunoprecipitated and assessed for their ability to phosphorylate exogenously added recombinant GST-STAT3 in an *in vitro* kinase assay with and without ATP. Data are representative of three independent repeats.

(B) Expression analysis of GFP-SteE in WT or *GSK3α/β*$^{-/-}$ 293ET cells in lysates (L) or depleted lysates (DL) after anti-GFP immunoprecipitation. This figure corresponds to the mass spectrometry data shown in Figure 4C.

(C) Nucleotide sequence prior to the annotated start GTG (bold) for STM2585, uniprot# Q8ZN17 ("Long" form) and including the start ATG (green) for the "Short" form with the consensus Shine-Dalgarno sequence underlined. Immunoblot analysis of *steE* mutant *Salmonella* carrying either no plasmid (-), control plasmid (pCon), or the long (L) or short (S) forms of SteE:HA following *in vitro* growth of *Salmonella* at pH 5.

(D) Luciferase activity in cell lysates from 293ET cells co-transfected with plasmids encoding a STAT3-dependent *Firefly* luciferase, a constitutively expressed *Renilla* luciferase and GFP or the indicated GFP-SteE variant. Data are presented as the fold change in STAT3 reporter activity from GFP-expressing cells and represent the mean and SEM of four independent experiments. Statistical significances were calculated from wild-type GFP-SteE. * $P < 0.05$, *** $P < 0.001$, one-way ANOVA with Dunnett's post hoc analysis for multiple comparisons. A representative anti-GFP immunoblot is shown for each expressed construct.

Figure S6, related to Figure 5: Analysis of STAT3 phosphorylation by GSK3

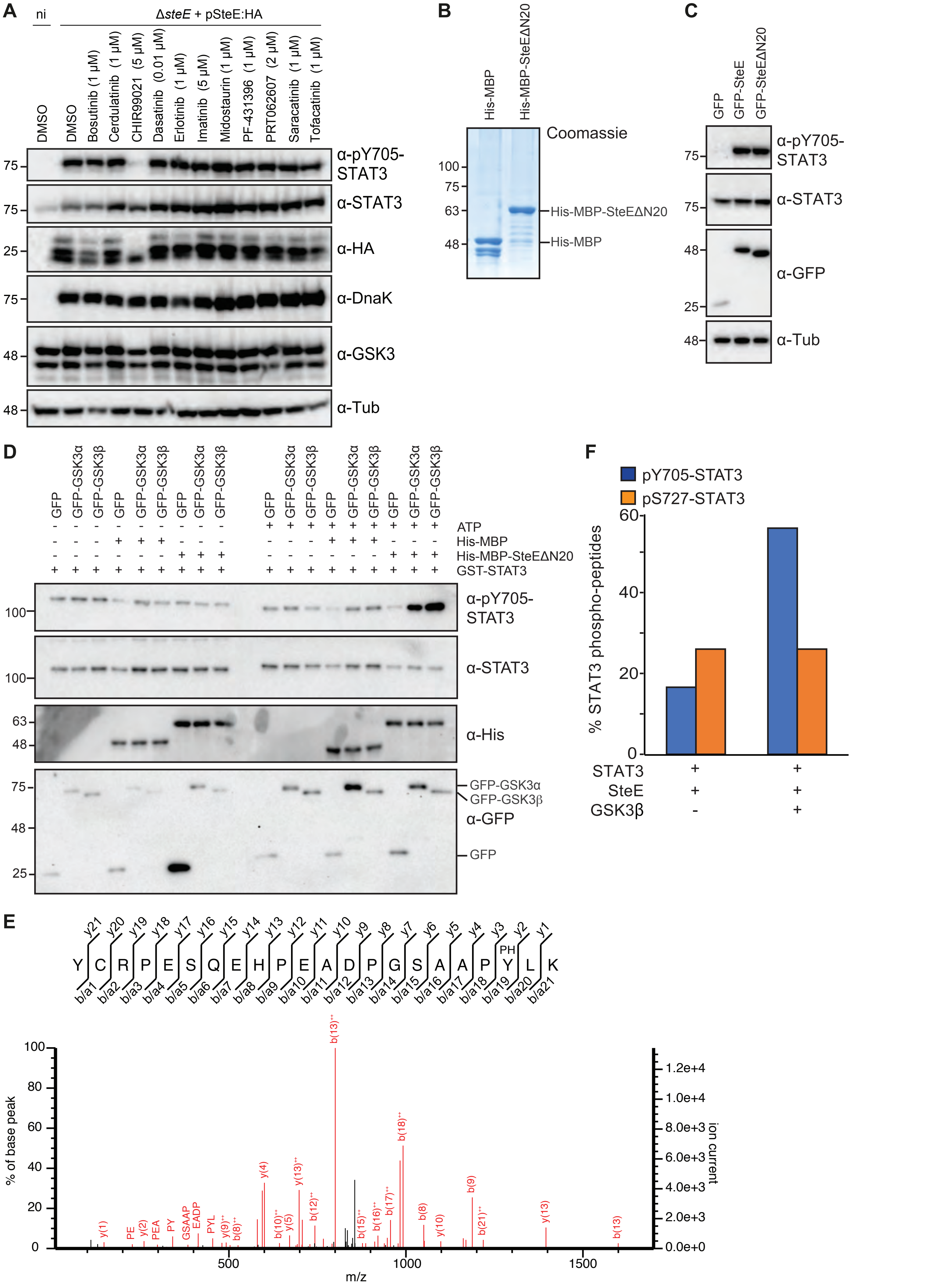

Figure S6, related to Figure 5: Analysis of STAT3 phosphorylation by GSK3

(A) WT 293ET cells were either non-infected (ni) or infected with *steE* mutant *Salmonella* carrying pWSK29-SteE:HA for 17 hours and treated with DMSO or the indicated drug at 1 hour post infection. Whole cell lysates were analysed for STAT3 and pY705-STAT3, as well as HA (SteE), DnaK (*Salmonella*) and tubulin (Tub) as a loading control. Data are representative of two independent repeats.

(B) Coomassie of recombinant protein used in *in vitro* kinase assays. His-MBP or His-MBP-SteEΔN20 were expressed in *E. coli* and purified on nickel beads.

(C) GFP, GFP-SteE or GFP-SteEΔN20 was transiently expressed in 293ET cells and cell lysates were then immunoblotted for STAT3, pY705-STAT3, GFP and tubulin (Tub) as a control.

(D) Cell lysates from 293ET cells stably expressing GFP, GFP-GSK3α or GFP-GSK3β were prepared and GFP immunoprecipitated proteins were used in *in vitro* kinase assays containing 0.4 μg recombinant GST-STAT3 and either buffer alone, recombinant His-MBP or recombinant His-MBP-SteEΔN20, with and without 1 mM ATP. Protein expression, and STAT3 phosphorylation were then analysed by immunoblot with the indicated antibodies. Data are representative of two independent repeats.

(E) Recombinant STAT3 was incubated with recombinant SteE with or without recombinant His-GSK3β in an *in vitro* kinase assay and subjected to mass spectrometry. A representative fragment analysis of a STAT3 Y705 containing peptide from the GSK3β-positive sample is shown.

(F) The ratio of phosphorylated and non-phosphorylated STAT3 peptides containing Y705 or S727 from the samples described in (E) are shown. Data is obtained from two independent experiments.

Table S1. PCR primers related to Key Resource Table

Plasmid	Sequence (5’ → 3’)	Forward/ Reverse	Restriction site	Purpose
pWSK29.ssaG	CATGGAATTCCTTGTGGTTTT CCTTAGGAGGTA	Forward	EcoRI	Amplification of SteE (short) + 25 bp Shine-Dalgarno sequence
	CATGGAATTCGCATGTAAAAG GGTCTCCTCTT	Forward	EcoRI	Amplification of SteE (long) + 25 bp Shine-Dalgarno sequence
	CATGGGATCCTTCATCCGGG AAAACCTCTGC	Reverse	BamHI	Amplification of SteE (short/long)
ptCMV/m4p/m6p	CGCGGGCCATGGCAATGTTT ACAATTAATAGTACTAA	Forward	NcoI	Amplification of SteE (short)
	CGCGGGGCGGCCGCTTATTC ATCCGGGAAAACCTCTG	Reverse	NotI	Amplification SteE
	CGCGGGCTCATGACAATGAG CGGCGGCGGGCCTTCGGGA	Forward	BspHI	Amplification GSK3α
	CGCGGGGCGGCCGCTCAGG AGGAGTTAGTGAGGGTA	Reverse	NotI	Amplification of GSK3α
	CGCGGGACATGTCAGGGCG GCCCAGAACC	Forward	PciI	Amplification of GSK3β
	CGCGGGGCGGCCGCTCAGG TGGAGTTGGAAGCTGATG	Reverse	NotI	Amplification of GSK3β
	CATGGCGGCCGCTCAGGCAG TTGGTGTATACTCC	Reverse	NotI	Amplification of GSK3β (1-327)
	CATGGCGGCCGCTCACCCTG GAAATATTGGTTG	Reverse	NotI	Amplification of GSK3β (1-259)
	CATGGCGGCCGCTCAAGGAT CCAACAAGAGGTTC	Reverse	NotI	Amplification of GSK3β (1-191)
	CATGGCGGCCGCTCAATAGT CCAGCACCAGATTAAG	Reverse	NotI	Amplification of GSK3β (1-134)
	CATGACATGTCATATACAGAC ACTAAAGTGATTGGAAATG	Forward	PciI	Amplification of GSK3β (56-420)
ptCMV/m4p/m6p	CATGACATGTCATATGTTCCG GAAACAGTATACAG	Forward	PciI	Amplification of GSK3β (134-420)
	GACCAGGGAACTAGTCGCCA TCGCGAAGGTTCTCCAGGAC AAGAGG	Forward	Internal primer	Amplification of GSK3α K148A
	CCTCTTGTCCTGGAGAACCTT CGCGATGGCGACTAGTTCCC TGGTC	Revrse	Internal primer	Amplification of GSK3α K148A
	CAGGAGAACTGGTCGCCATC GCGAAAGTATTGCAGGACAA GAGA	Forward	Internal primer	Amplification of GSK3β K85A
	TCTCTTGTCCTGCAATACTTT CGCGATGGCGACCAGTTCTC CTG	Reverse	Internal primer	Amplification of GSK3β K85A
	AATCTGGTGGGTGAATATGT GCCCGAGACA	Forward	Internal primer	Amplification of GSK3α L195G
	CACATATTCACCCACCAGATT TAGGTAAAG	Reverse	Internal primer	Amplification of GSK3α L195G
	GGATTACAGGAACGTATAGC ACTCGAGTACCAGCCCCTG	Forward	Internal primer	Amplification of SteE S76A
	CAGGGGCTGGTACTCGAGTG CTATACGTTCCTGTAATCC	Reverse	Internal primer	Amplification of SteE S76A
	ATTGTTTTTCTACTCGGCGCG CCTGCAGTTTTAGAGACT	Forward	Internal primer	Amplification of SteE T91A

	AGTCTCTAAAACTGCAGGCGCGCCGAGTAGAAAAACAAT	Reverse	Internal primer	Amplification of SteE T91A
	TCTTTATCATTACCAGTTGCGCCGGATGCTTTAACCCAA	Forward	Internal primer	Amplification of SteE S106A
	TTGGGTTAAAGCATCCGGCGCAACTGGTAATGATAAAGA	Reverse	Internal primer	Amplification of SteE S106A
	CGCGGGGCGGCCGCTTATTCATCCGGGAAAACCTCTGCAGAATGCCTGTATTGAGCGATATAACCAGCCGGTGGGTTATGACTGGC	Reverse	NotI	Amplification of SteE S141A
	CGCGGGGCGGCCGCTTATTCATCCGGGAAAACCTCTGCAGAATGCCTGTATTGAGCGATAAAACCGGACGGTGGGTTATGACTGGC	Reverse	NotI	Amplification of SteE Y143F
	CGCGGGGCGGCCGCTTATTCATCCGGGAAAACCTCTGCAGAATGCCTGTATTGAGCGATAAAACCAGCCGGTGGGTTATGACTGGC	Reverse	NotI	Amplification of SteE S141A and Y143F
**m6p**	CGCGGGACATGTCAGATGTTAATTTAGAGGAC	Forward	PciI	Amplification of SteE (short) ΔN20
	GAGCTGTACAAGGACATGACAATGAGCGGCGG	Forward	Gibson cloning	Amplification of GFP-GSK3α
	TCCGGATCTGTTAACGCGGCCGCTCAGGAGGAGTT	Reverse	Gibson cloning	Amplification of GFP-GSK3α
**pET49**	CCTCTTTCAGGGACCCGGTAGCGATGTTAATTTAGAGGAC	Forward	Gibson cloning	Amplification of SteE ΔN20
	CCGCGTGGCACCAGAGCGTTATTATTCATCCGGGAAAACCTCTG	Reverse	Gibson cloning	Amplification of SteE ΔN20
**pACEBac**	GGGCGCGGATCCCGGTATGTCTAGTGGTTCTGGTCATCACCATCAC	Forward	Gibson cloning	Amplification of GSK3β
	TTTGAATTCCGCGCGCTTCGGTCAGGTGGAGTTGGAAGCTGATG	Reverse	Gibson cloning	Amplification of GSK3β
	GGGCGCGGATCCCGGTATGAAACATCACCATCACCATCACCC	Forward	Gibson cloning	Amplification of STAT3
	TTTGAATTCCGCGCGCTTCGGTCACATGGGGGAGGTAGCGCACTCCG	Reverse	Gibson cloning	Amplification of STAT3
